# Generation of Bloch Points with Controlled Spin Texture Using Geometrical Boundary Conditions

**DOI:** 10.1021/acsnano.6c09658

**Published:** 2026-10-13

**Authors:** Naëmi Leo, Daniel Wolf, Alicia Estela Herguedas-Alonso, Oleksandr Zaiets, Jakub Jurczyk, Takeaki Gokita, John Fullerton, Dedalo Sanz-Hernandez, Claire Donnelly, Andrea Sorrentino, Eva Pereiro, Lucia Aballe, Peter Fischer, Rachid Belkhou, Claas Abert, Dieter Suess, Axel Lubk, Aurelio Hierro-Rodriguez, Amalio Fernández-Pacheco

**Affiliations:** † Department of Physics, 5156 Loughborough University, Loughborough LE11 3TU, UK; ‡ Institute of Applied Physics, 27259 TU Wien, Vienna 1040, Austria; § Leibniz Institute for Solid State and Materials Research Dresden, Helmholtzstr. 20, Dresden 01069, Germany; ∥ Departamento de Física, 16763 Universidad de Oviedo, Oviedo 33007, Spain; ⊥ CINN (CSIC—Universidad de Oviedo), El Entrego 33940, Spain; # 430128 ALBA Synchrotron Light Source, Cerdanyola del Valles, Barcelona 08290, Spain; ∇ Institute of Solid State and Materials Physics, Technische Universität Dresden, Dresden 01069, Germany; ○ Materials Science Division, 1291 Argonne National Laboratory, Lemont, Illinois 60439 United States; ◆ 131692 Laboratoire Albert Fert, CNRS, Thales, Palaiseau 91767, France; ¶ 28270 Max Planck Institute for Chemical Physics of Solids, Dresden 01187, Germany; ◮ Materials Sciences Division, 1666 Lawrence Berkeley National Laboratory, Berkeley, California 94720, United States; ◭ Physics Department, University of California Santa Cruz, Santa Cruz, California 95064, United States; 7 360818 Synchrotron SOLEIL, Saint-Aubin 91190, France; 8 Faculty of Physics, 55536 University of Vienna, Vienna 1090, Austria

**Keywords:** bloch points, nanomagnetism, electron holography tomography, magnetic soft xray tomography, magnetic chirality, helical nanowires

## Abstract

Bloch points are three-dimensional topological singularities in magnetization that play a key role in topological transformations of spin textures such as skyrmion creation or annihilation. While topology often enforces the existence of Bloch points in confined geometries such as cylindrical nanowires, deterministic control over their position and magnetic configuration remains challenging. Here, we demonstrate the generation of Bloch points with controlled spin texture by engineering geometrical boundary conditions in three-dimensional nanomagnets. By introducing a chirality interface between two three-dimensional double-helix nanowires of opposite handedness, forming a kinked, noncollinear structure, we impose competing topological constraints that uniquely define the magnetization configuration surrounding the Bloch point. A saturating magnetic field nucleates head-to-head or tail-to-tail domain configurations at the chirality interface, producing a Bloch-point domain wall with deterministic polarity, circulation, and helicity. This geometrical approach enables full three-dimensional control of Bloch point domain walls, allowing deterministic engineering of their spin texture and its selective coupling to current-induced Oersted fields.

## Graphical abstract

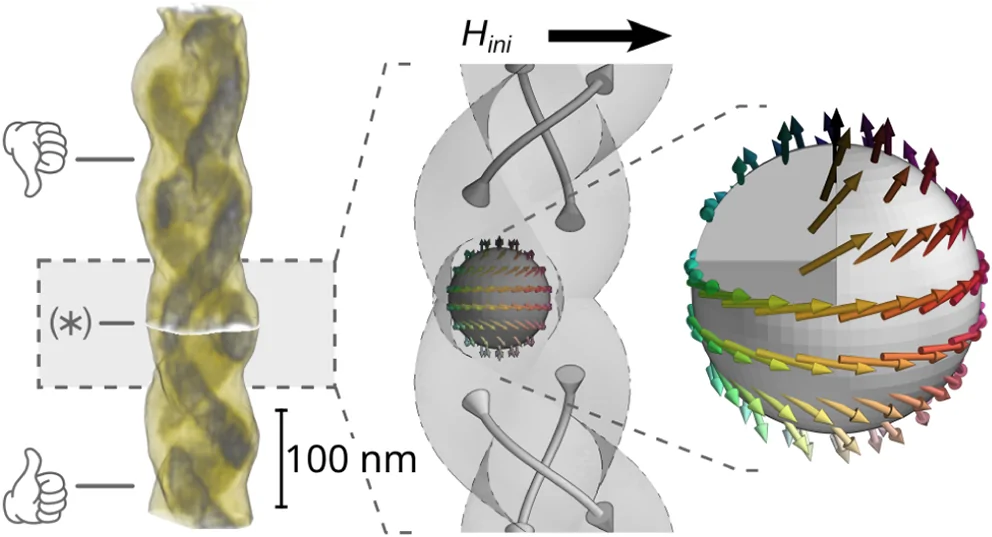

## 1 Introduction

Topological defects play a central role in magnetism, governing both the stability and dynamics of nanoscale magnetic textures. Among them, Bloch points are true three-dimensional singularities of the magnetization field, where the magnetization vanishes and its direction becomes undefined.^1–7^ Bloch point singularities arise in a wide range of magnetic systems and are key to topological features of certain magnetic solitons, including chiral bobbers,^8,9^ interfaces between skyrmion tubes of different topology,^10,11^ Bloch point domain walls,^12–14^ and screw dislocations in helimagnets.^15^ Bloch points also play a crucial role in a wide range of transient magnetization processes,^16,17^ such as the mobility of the aforementioned solitons,^9^ magnetic vortex core reversal,^18^ and the change of topological charge during the creation and annihilation of vortex-antivortex pairs and skyrmions.^9,19^ By providing a transient singular configuration of the magnetization field, Bloch points enable topological transitions that are otherwise prohibited by the topological stability of these textures.

Although the influence of Bloch points on the field of nanomagnetism has been recognized for decades, only recent advances in nanoscale magnetic imaging have allowed us to experimentally probe their 3D spin order^20,21^ and behavior under applied fields or current pulses.^22,23^ Regardless of this progress, the experimental understanding of predicted dynamical properties of Bloch points^3,13,14,19,24^ and their use for potential spintronic functionalities^4,11,25–27^ is still hampered by the difficulty to control their nucleation, including both their spatial position and their magnetic configuration surrounding the singularity. In particular, it remains an open question whether the spin texture around a Bloch point can be deterministically defined through designed materials and geometrical engineering, rather than emerging stochastically from symmetry or defects.

In this work, we demonstrate deterministic control of Bloch point nucleation by engineering geometrical boundary conditions, implemented via a chirality interface between 3D-printed helical nanowires. Using advanced nanotomography techniques with magnetic sensitivities, including transmission electron microscopy (TEM) and magnetic soft X-ray transmission microscopy, supported by micromagnetic simulations, we confirm that domain walls containing circulating Bloch points are reliably nucleated with predefined polarity, circulation, and helicity angle. By imposing competing geometrical constraints at the interface, the spin texture surrounding the Bloch point can be uniquely defined. This approach, combining tailored geometry and a robust field initialization protocol, provides a pathway toward 3D spintronic device architectures containing Bloch points.

## 2 Bloch Point Singularities

The micromagnetic state in the vicinity of an axially symmetric Bloch point can be described by an analytical model^28,29^ using spherical coordinates, expressing the local direction of the reduced moment **m**(**r**) = (*m* = 1, θ_*m*_, ϕ_*m*_) at position **r** = (*r*, θ, ϕ):

1 $$\theta_{m}\left(\theta \right)=p\theta +\frac{\pi }{2}\left(1-p\right)$$

2 $$\phi_{m}\left(\phi \right)=v\phi +\gamma$$

Here, the discrete parameters *p*, *v*, and the continuous angle γ are key quantities that determine the static and dynamic properties of the Bloch point.^28^ The discrete polarity *p* determines whether a tail-to-tail (←→, *p* = +1) or head-to-head (→←, *p* = –1) configuration is observed when crossing the equatorial plane. The discrete vorticity *v* determines whether the spin state within the equatorial plane is a vortex (*v* = +1) or an antivortex state (*v* = –1). Finally, the continuous helicity angle γ describes how moments at the equator point either outwards (γ = 0°) or inwards (γ = 180°) for a hedge-hog-like Bloch point, or rotate around the central axis (0° < |γ|< 180°) in a twisted Bloch point configuration.

A subset of twisted Bloch points are the four vortex-like *circulating* Bloch points depicted in Figure 1a–d, for which *v* = +1, *p* = ±1, and γ = ±90°. When viewed from above, these Bloch points exhibit a well-defined counterclockwise [ccw, γ = +90°, Figure 1a,c] or clockwise [cw, γ = −90°, Figure 1b,d] circulation in their equatorial plane. This circulation sense is degenerate in highly symmetric systems. For instance, in cylindrical nanowires, clockwise and counterclockwise Bloch points appear with equal probability.^16,17,30^ A uniformly chiral geometry (e.g., a helix with fixed handedness) likewise does not uniquely select the circulation of a Bloch point, since both clockwise and counterclockwise configurations remain equivalent. This reflects the fact that a Bloch point connects regions of opposite magnetic chirality: as shown in Figure 1c, the magnetic chirality χ_*M*_—which can be understood as the product between the sense of circulation and the sign of the axial magnetization (also called vortex polarization in ref 31)—of the top (χ_*M*_ right-handed, RH) and bottom half (χ_*M*_ left-handed, LH) of the spin state cancel each other out, i.e., $\chi_{M}^{\mathrm{top}}=-\chi_{M}^{\mathrm{bottom}}$ , i.e., the net Bloch point spin texture is achiral. Deterministic selection is only achieved when chirality is imposed *locally*, for example at an interface that breaks the chirality equivalence.

**Figure 1:**
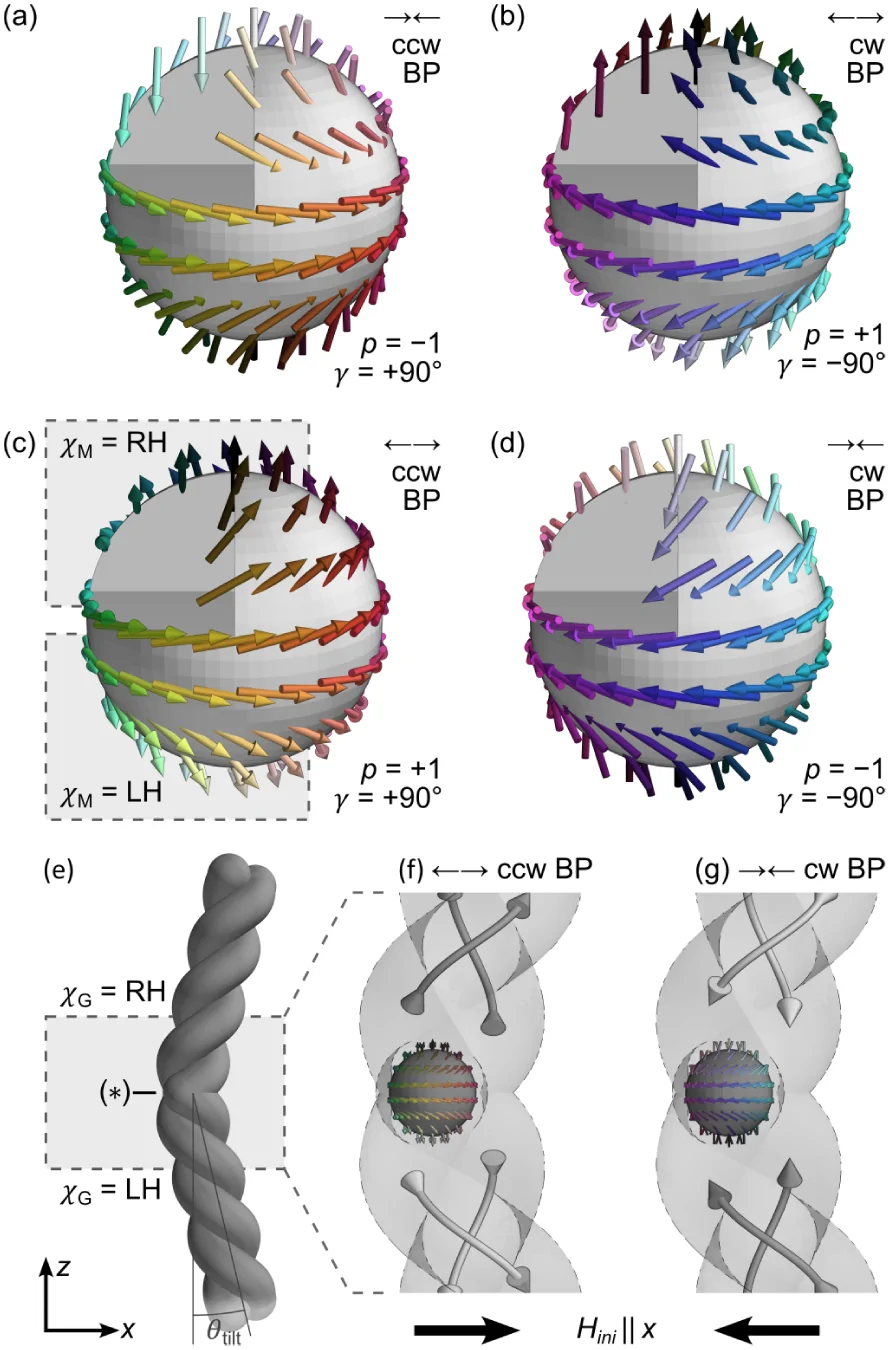
Geometrical control of Bloch point spin texture using a chirality interface. (a–d) Circulating Bloch points with varying polarity *p* (head-to-head →←, *p* = −1 and tail-to-tail ←→, *p* = +1) and helicity γ describing clockwise (cw, γ = −90°) and counterclockwise (ccw, γ = +90°) spin circulation. These parameters define the spin state around the Bloch point singularity. While each half of the spin texture around a Bloch point has a well-defined magnetic chirality χ*_M_*, as shown in (c), the net Bloch point spin texture is achiral. (e) 3D nanowire connecting a left-handed (LH, bottom) with a right-handed (RH, top) double helix, with each segment tilted by ±θ_tilt_ away from the *z* direction. The chirality interface is marked with (*) and imposes competing geometrical boundary conditions. (f,g) Field-induced initialization of Bloch points after saturation with a sufficiently strong field **H**_ini_||*x*. The sign of *H*_ini_ sets the Bloch point polarity, while its circulation is determined by the geometrical chirality χ*_G_* on either side of (*), resulting in Bloch points with controlled spin texture.

Tailored helical nanostructures grown by recently established 3D nanoprinting techniques^32–35^ enable interfacing opposing geometric chirality regions for the controlled nucleation of circulating Bloch points. As shown in previous works,^31,36^ such double-helix nanowires allow to control the magnetic chirality through their geometric chirality, i.e., χ_*M*_ = χ_*G*_, thereby enabling the controlled creation of metastable spin structures such as chiral domain walls^36^ and skyrmion tubes^31^ by simple magnetic-field protocols. Here, we extend this geometric-control approach by deliberately modifying the angle between chiral segments of the double helix and investigating the resulting spin configurations under more complex magnetic-field protocols, enabling robust field-induced nucleation of Bloch-point domain walls with controlled polarity, circulation, and helicity.

The proposed structure for controlled Bloch point nucleation is shown in Figure 1e and consists of double helices of opposite geometric chirality χ_*G*_, here a left-handed segment at the bottom and a right-handed segment at the top, meeting at the chirality interface marked with (*). Each segment is tilted by an angle of ±θ_tilt_ away from the longitudinal *z*-axis. This tilt toward the *x*-axis allows initialization of a head-to-head or tail-to-tail domain wall at the chirality interface with a sufficiently strong transverse magnetic field **H**_ini_||*x*.^30^ Subsequent relaxation of the spin structure, together with the imprinted opposing geometric chirality on both sides of the interface, supports the stabilization of a central circulating Bloch point at remanence.

Importantly, there is a direct coupling between the magnetostatic charge and the circulation of the created Bloch point, determined by the change of geometric chirality at (*) and by the sign of *H*_ini_ in this system. In our case, interfacing left- and right-handed double helices stabilizes either a tail-to-tail counterclockwise or a head-to-head clockwise Bloch point domain wall, as shown in Figure 1f,g. Therefore, this geometrical interface-induced mechanism enables simultaneous control over both the Bloch point polarity (head-to-head or tail-to-tail, set by the direction of the initially applied field), its circulation (set by the RH/LH or LH/RH order of the helical segments), and its position (localized close to the chirality interface). This level of control is not achievable in cylindrical nanowires, where the position of Bloch points can be influenced by curvature,^27^ yet both the tunability of its spin texture and spatial determinism remain limited.^16,17,30^ The key enabling factor here is the connectivity of two double-helix nanowires via the chirality interface (*), a topological structural defect, which imprints opposite geometric chirality χ_*G*_ across the interface and thus a corresponding opposite magnetic handedness χ_*M*_.

## 3 Results

### 3.1 Tailoring the Geometric Chirality Interface

We used focused-electron beam-induced deposition (FEBID)^35^ to 3D nanoprint free-standing magnetic Co helical nanowires^31,36^ (Section 5.1). Scanning electron microscopy (SEM) images in Figure 2a,b show the resulting structures in side and front views. In the middle of the structure, marked with (*), the geometric chirality of the double helix sections changes from $\chi_{G}^{\mathrm{bottom}}=\mathrm{LH}$ to $\chi_{G}^{\mathrm{top}}=\mathrm{RH}$ . Each chiral section is inclined by θ_tilt_ = ±15°, with the value of the tilt angle being a compromise of three considerations: First, 15° is sufficiently large for a transverse field *H*_*x*_ to initialize the head-to-head or tail-to-tail configuration required to stabilize a circulating Bloch point. Second, considering FEBID growth, larger tilt angles result in overhangs that are difficult to realize and, furthermore, promote electron scattering that can lead to undesirable codepositions. Third, as indicated by micromagnetic simulations, a tilt angle of 15° is small enough to suppress flux leakage at the chirality interface, which would favor the formation of a transverse domain wall instead of a Bloch point domain wall.

**Figure 2:**
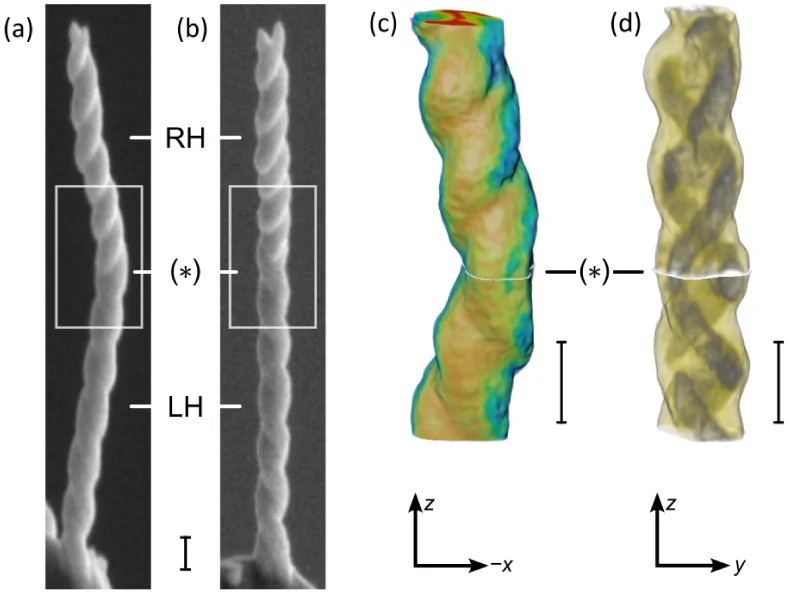
3D nanostructure defining the geometrical boundary conditions for Bloch point generation. (a) Side and (b) front view of FEBID-grown Co double-helix structures, combining a bottom LH and top RH section tilted by ±15°. (c) Opaque and (d) transparent volume rendering of the nanowire’s mean inner potential (proportional to material contrast) reconstructed by electron holographic tomography. The position of the chirality interface is marked by (*) and defines the boundary where Bloch points are nucleated and their topology is imposed. Scale bars measure 100 nm.

We performed TEM-based off-axis electron holographic tomography, a combination of electron holography and electron tomography, to obtain a three-dimensional reconstruction of the electrostatic potential^37,38^ (Section 5.3). The electrostatic potential arises from the averaged atomic potentials of the material over a volume of a few cubic nanometers (for crystalline materials referred to as mean inner potential) and thus can be used to visualize the material contrast and the 3D morphology of the nanostructure.

Figure 2c shows the 3D morphology of the double-helix structure in the vicinity of the chirality interface, rendered with cubic voxels with an edge length of 0.8 nm. The nanostructure shape revealed closely resembles the CAD geometry used for micromagnetic simulations shown in Figure 1e. The chirality interface at (*), where the bottom LH helix merges into the top RH helix, can be easily identified and is marked with a white outline. Figure 2d depicts a transparent 3D volume highlighting the inner structure of the nanowire in gray to emphasize the interwoven strands deposited during the layer-by-layer 3D printing process. A 3D animation and further information are provided in the Supporting Information.

A reduced diffraction contrast observed in bright-field TEM images (not shown) suggests that individual Co grains are typically smaller than 10 nm, as previously reported for this FEBID material.^31^ The obtained nanocrystalline structure indicates that the magnetic anisotropy primarily arises from the imprinted nanowire geometry. This shape anisotropy is crucial because it establishes the strong coupling between magnetic and geometric chirality, i.e., χ_*M*_ = χ_*G*_.

### 3.2 Direct Observation of Bloch Points by 3D Magnetic Tomography

To directly demonstrate the existence of Bloch points in our system, we employ magnetic soft X-ray nanotomography at the MISTRAL beamline of the ALBA synchrotron.^39^ Using the X-ray magnetic circular dichroism (XMCD) signal from the Co *L*_2_ absorption edge, which measures the projection of the magnetization onto the photon propagation direction, and different sample orientations, we can obtain a volume-resolved reconstruction of the vector character of the local magnetic moments. As a result, the measurements provide direct access to the three-dimensional spin texture surrounding the Bloch point down to the spatial resolution limit of about 50 nm.

The Bloch point state was initialized by applying a transverse field of about 1 T along the kink of the nanowire, following the scheme described in Figure 1f–g (see Section 5.2). To perform a vector magnetic tomographic reconstruction,^20,40^ the results of two measurement series were combined—one, where the double-helix nanostructure is rotated around its main axis from −65° to +64° in 1.5° steps, as well as getting tilted toward the beam from −33° to +19° in 1° steps (see Section 5.2 and Supporting Information Section 1.2 for further details).

Figure 3a shows representative XMCD measurements for the rotation (horizontal images) and tilt (vertical images) series. From the highlighted XMCD images in Figure 3a, one can deduce: (i) a magnetic state of uniform circulation throughout the nanostructure and (ii) opposite axial magnetization at the two sides of the chirality interface, i.e., for the two segments of opposite geometric chirality. Figure 3b shows the reconstructed structural contrast of the double helix geometry with the central kink corresponding to the geometrical chirality interface highlighted by a white plane. Analyzing the magnetization reconstruction in the vicinity of the chirality interface (Figure 3c), we confirm the presence of a head-to-head Bloch point with clockwise circulation (when viewed from above), in agreement with our expectations from Figure 1g.

**Figure 3:**
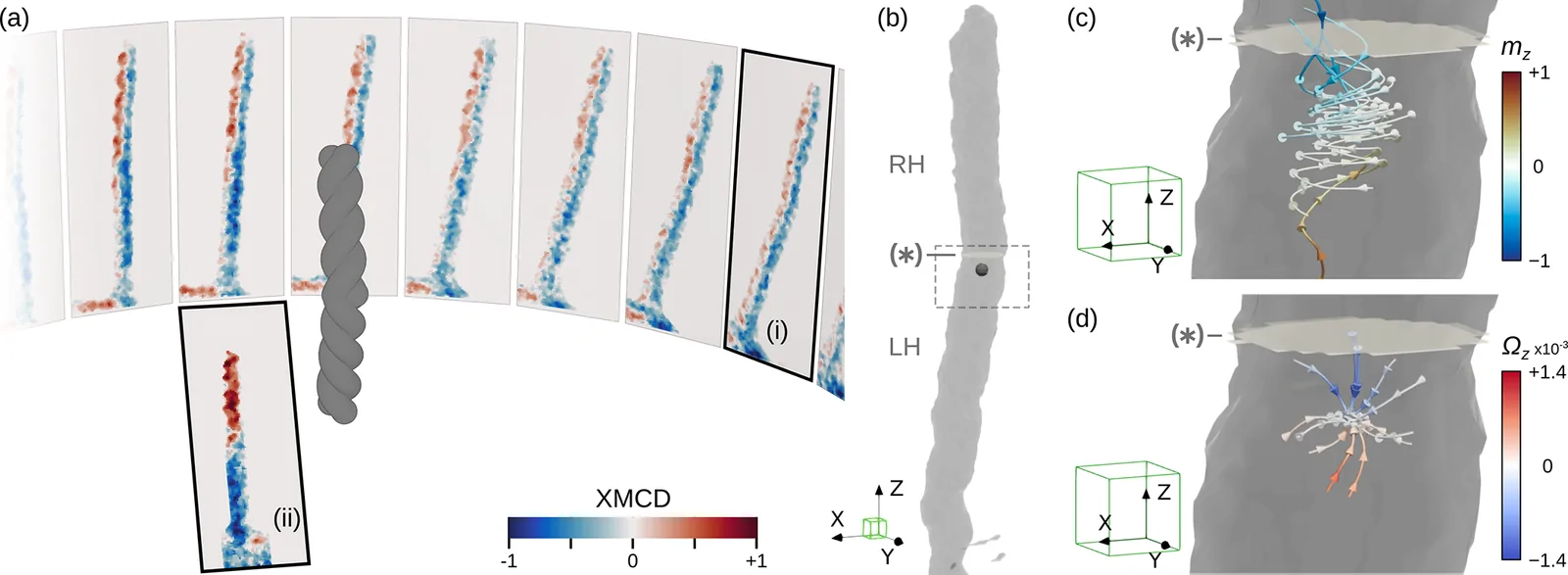
Direct observation of Bloch points with controlled spin texture by XMCD tomography. (a) Selection of XMCD images taken under different rotation and tilt angles for the reconstruction of the 3D magnetization. The XMCD contrast in the highlighted panels indicates (i) uniform circulation across the length of the nanostructure and (ii) opposite axial magnetization in both helical segments, implying the presence of a Bloch point. (b) Reconstructed electronic structure with a voxel size of (10 nm)^3^. The chirality interface plane is highlighted with (*). The region of interest around the Bloch point (gray sphere) shown in (c,d) is highlighted (dashed line). (c) Reconstructed magnetization **m**_sol_(**r**) of a clockwise-circulating head-to-head Bloch point, directly revealing a Bloch point with well-defined polarity and circulation. (d) Magnetic vorticity field **Ω**(**r**), showing that the Bloch point acts as a sink for the emergent field and confirming its topological character. Scale cubes have an edge length of 50 nm. Streamlines and arrows denote the local direction of the vector fields **m**(**r**) and **Ω**(**r**), and the color denotes the magnitude of the axial components *m_z_* and Ω*_z_*, respectively.

Interestingly, the singularity position, marked with a black sphere in Figure 3b, is offset by about 60 nm from the chirality interface [(*), white plane] toward the bottom left-handed segment. Recent results have shown that Bloch point domain walls are energetically unfavorable in sections of high geometric curvature.^27^ Here, the kink introduces a localized curvature maximum at the chirality interface and therefore is expected to play an important role in the energetic landscape and pinning of the spin texture around the Bloch point.

Considering the spin dynamics governed by the damping term in the Landau–Lifschitz–Gilbert (LLG) equation, the displacement of a Bloch point domain wall from a region of high curvature is predicted to depend on the Bloch point polarity (see Supporting Information Section S2). Specifically, analytical arguments and micromagnetic simulations show that upon initialization from a saturated state, a head-to-head (tail-to-tail) Bloch point moves toward the left-handed (right-handed) chiral section. The displacement observed in the XMCD data agrees with that of this prediction. However, as shown below by the higher-resolution TEM measurements, the predicted curvature-driven polarity-dependent displacement may not be applicable in nonideal nanostructures, and the experimentally observed Bloch-point position might be dominated by other factors such as local pinning sites, sample-to-sample microstructural variations, and details of the applied field protocol.

The displacement of the Bloch point from the chirality interface at (*) also has important consequences for the local magnetic configuration, as it creates a small segment in which the condition χ_*M*_ = χ_*G*_ is not fully obeyed. As we previously discovered,^31^ in this segment, a metastable fractional skyrmion-like tube state is stabilized. Here, this spin texture terminates at the Bloch point.

In Figure 3d we represent streamlines of the magnetic vorticity **Ω**(**r**)^41^ calculated from the reconstructed magnetization (eq 5). The vorticity **Ω**(**r**) has the same functional form as the emergent (topological) magnetic field and differs only by a constant prefactor. For the head-to-head magnetic configuration observed here, the Bloch point acts as a sink for the emergent field, as indicated by the arrows on the streamlines of **Ω**(**r**) in Figure 3d, whereas a tail-to-tail Bloch point would act as a source. This source–sink behavior reflects the effective monopole-like character of the Bloch point in the emergent field and its associated nontrivial topology.

Finally, the XMCD vector reconstruction allows us to estimate the helicity angle γ in eq 2, providing access to the third parameter describing the spin texture around a Bloch point. From the experimental data, we found γ ≈100°, in good agreement with simulations (Supporting Information Section S3). This value is set by the local geometry of the system, in this case the kinked double helix with a chirality interface. The value γ > 90° means that spins in the equatorial plane of the Bloch point not only circulate but also twist inward, creating a Bloch point of slightly hyperbolic character.^29^

### 3.3 High-Resolution Magnetic Imaging by Electron Holography

Having established the presence of Bloch points through direct XMCD tomography, we now employ electron holography and electron holographic tomography to obtain higher resolution insight into the magnetic structure at the chirality interface. To this end, we investigate a similar nanostructure as before, initializing the Bloch point with a multistep field protocol within the transmission electron microscope, while the experimental protocol follows the same principle as the initialization shown in Figure 1f,g and used in the XMCD experiments (Section 5.3.2). Electron holographic tomography reconstructs, in addition to the electrostatic potential, the three-dimensional distribution of components of the magnetic induction **B** within^37,38^ and outside^42^ a 3D nanostructure at nanometer resolution. In a single-axis tomography experiment, we reconstructed the component of the magnetic induction parallel to the rotation axis only.

Figure 4a shows the combined experimental dataset of the external and internal magnetic state at multiple angles measured at remanence. These results are compared to micromagnetic simulations shown in Figure 4b (Section 5.4). Projections taken from different directions show electrostatic phase maps in gray superimposed with magnetic equiphase lines in green representing magnetic field lines transversal to the electron beam direction. A volume rendering in the center shows the reconstructed magnetic induction *B*_*z*_(**r**) within the nanostructure, indicating the head-to-head domain configuration with a blue-red contrast.

**Figure 4:**
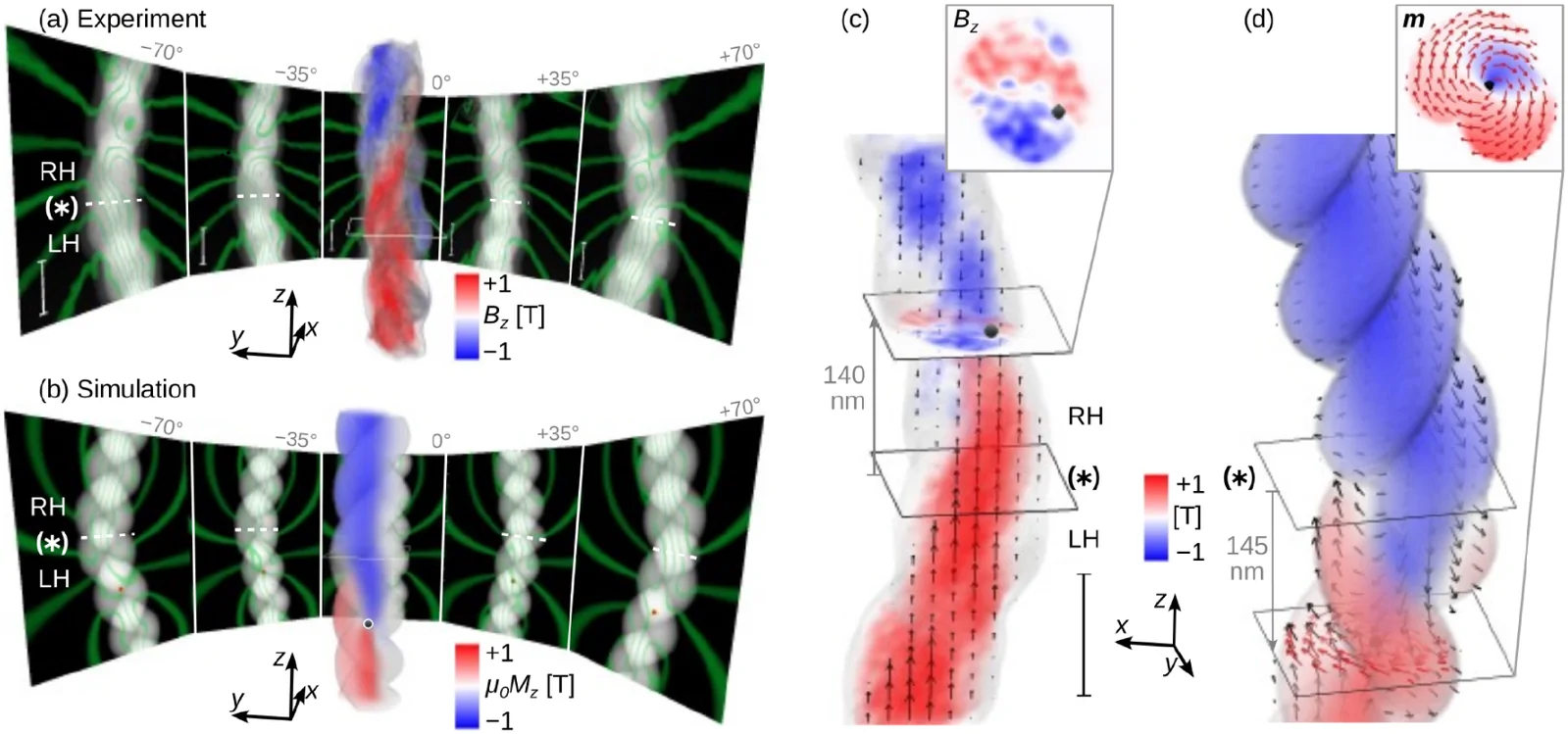
Stray-field and internal magnetic induction revealing details of Bloch point domain walls. (a) Experimental and (b) simulated phase images after initialization of a Bloch-point state, viewed under different projection angles. The electric and magnetic contributions are shown in grayscale and as green iso-lines, respectively. The chirality interface (*) is marked by dashed lines and cut planes. The monopole-like symmetry of the stray-field patterns is consistent with the presence of a Bloch point. (c) Tomographic reconstruction of the internal magnetic induction component *B_z_* (**r**) [also shown in the foreground of (a)], showing the domain wall associated with the Bloch point. The inferred position of the singularity is marked with a black dot (see inset plane). (d) Magnetization state **m**(**r**) obtained from micromagnetic simulations, with the inset showing the circulation in the equatorial plane of the Bloch point (black dot), confirming the circulating texture of the Bloch point. Scale bars measure 100 nm.

Both experimentally measured as well as simulated stray field lines show a high degree of symmetry with little change under different viewing angles. A directional variation of the far field would indicate leakage of magnetic flux, as is, for example, observed for transverse domain walls^43^ (see also Supporting Information Section S4 and Figure S6 for a comparison). The lack of variation in the field line pattern with the viewing direction therefore indicates a monopole-like far-field stray-field distribution, consistent with the presence of a Bloch point domain wall. Furthermore, for the simulated patterns shown in Figure 4b the field lines originate from the position of the Bloch point (indicated with a black sphere). The agreement between the measured and simulated datasets, together with the symmetry of the far-field lines, which indicates an underlying monopolar character, strongly supports the presence of a Bloch point in the experimental data, in full consistency with the XMCD tomography results discussed above.

Figure 4c,d compares the volume rendering of the internal induction *B*_*z*_(**r**) from the experimental reconstruction to the simulated magnetization state in greater detail. Red and blue indicate volumes where experimental *B*_*z*_ and simulated *m*_*z*_ point upward and downward, respectively, with a head-to-head domain wall in-between. The boundary between ±*B*_*z*_ volumes extends about 250 nm (i.e., about one helix periodicity) and is inclined in the nanowire (see also Supporting Information Figure S4).

Micromagnetic simulations in Figure 4d show comparable results to the experiment, as well as demonstrate a well-defined circulation across the length of the nanostructure (see insets). Furthermore, features in magnetic phase images computed from *m*_*z*_(**r**) simulations under certain angles, such as the antivortex-like textures shown in Figure 4b, can be inferred to be projections of the magnetic induction in the vicinity of the Bloch point. Since these features are also observed in experimental phase images, they can be used to triangulate the 3D position of the Bloch point from at least two different projection angles (). The Bloch point position is indicated with a black dot in the experimental data shown in Figure 4c. Along the axial *z* direction, the deduced Bloch point position also coincides with the *xy* plane where the average axial magnetic induction |⟨*B*_*z*_⟩_*xy*_| is minimal (see inset in Figure 4c,d). Inspecting the latter, we attribute the differences between the experimental and simulated cross sections to the fact that the position of the Bloch point is more off-center in the experiment than in the simulation.

The spin texture surrounding the Bloch point can be changed by a field initialization protocol. This is demonstrated in Figures 5, with [Fig fig5]a showing the visualized region around the chirality interface. Figure 5b–d represents the projected magnetic induction maps as arrow plots overlaid with green field lines and the axial *B*_*z*_ component of the nanowire shown in red-blue. Figure 5b and c compares the magnetic state obtained after initialization with opposite *H*_ini_. The change in red and blue contrast clearly demonstrates the formation of a head-to-head and tail-to-tail state, respectively. The circulation is not directly resolved due to the averaging over depth, but the good agreement with micromagnetic simulations, shown in Figure 5d, allows us to identify clockwise head-to-head and counterclockwise tail-to-tail Bloch point configurations, as predicted in Figure 1f,g.

**Figure 5:**
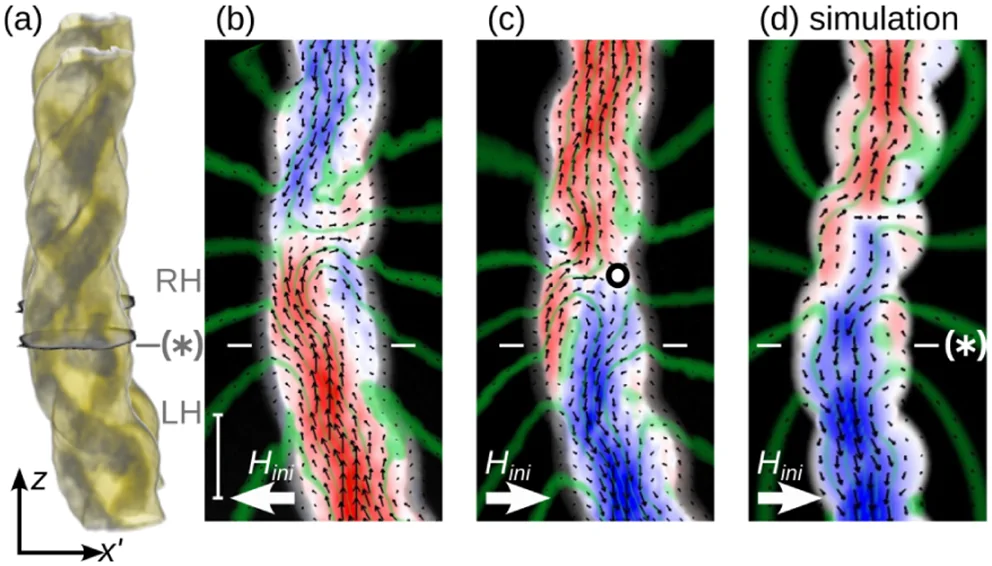
Deterministic control of Bloch point polarity and position. (a) Volume rendering of the structural contrast around the chirality interface (*). (b,c) Maps of external (green lines) and internal magnetic induction $\langle B_{x^{\prime }z}\left(x^{\prime },z\right)\rangle_{y^{\prime }}$ (arrows, blue/red denotes the magnitude of the *B_z_* component) at remanence, after initialization with opposite *H*_ini_, demonstrating controlled switching between head-to-head and tail-to-tail Bloch point configurations. (d) Equivalent induction map calculated from micromagnetic simulations, showing the state equivalent to (c). Inferred positions of the Bloch point are marked with black circles; these always show a displacement away from the chirality interface (*) due to curvature and pinning effects. Scale bar in (b) measures 100 nm.

In Figure 5b–c the Bloch point positions are marked with black circles, and in all cases the Bloch point is displaced along *z* away from the chirality interface (*) [dashed line] by 70 nm to 140 nm. Although there is overall good agreement with the micromagnetic simulations, we find a discrepancy in that the Bloch point is consistently pinned on the RH segment for all of the TEM measurements. This finding is in contrast to the above-discussed predicted behavior for the Bloch point to displace according to its polarity. We ascribe this discrepancy to pinning caused by the variation in density of magnetic Co (as indicated in Figure 2d, and in contrast with the homogeneous material assumed in the micromagnetic simulations), as well as possible field misalignments during the multistep Bloch point initialization procedure in the TEM experiments (Section 5.3.2).

In addition to an axial displacement away from the chirality interface, the Bloch point is also found to shift within the perpendicular (*x*, *y*) plane toward the outer surface of the structure. This transverse displacement can be related to competing micromagnetic energy contributions: while magnetostatic energy favors configurations that minimize stray fields and thus a more centered position, exchange energy penalizes the strong magnetization gradients associated with the Bloch point. In the curved geometry of the system, these gradients can be partially relieved by shifting the Bloch point toward the outer surface, leading to an off-center equilibrium position. Cobalt density variations (Figure 2d) may further favor the displacement of the Bloch point toward the surface. However, although these small variations in the reconstructed electrostatic potential indicate local microstructural inhomogeneities in the FEBID-grown Co nanowire, the structure is continuously grown and shows no obvious discontinuity at the chirality interface. We therefore interpret the axial Bloch point displacement primarily as a curvature-induced effect, while small local variations in the magnetic Co density may contribute to pinning and to deviations from the idealized homogeneous-material simulations. The superior spatial resolution of TEM holography therefore allows us to discern effects of the local structural environment on the location and behavior of a Bloch point.

The combined use of X-ray magnetic tomography, electron holographic tomography, and micromagnetic simulations allows us to probe the Bloch-point spin texture across different length scales, yielding a consistent three-dimensional picture of the magnetization around the Bloch point.

## 4 Conclusions

Bloch points—three-dimensional singularities where the magnetization vanishes—represent unique magnetic topological defects.^1,2^ However, strategies for their controlled nucleation have only recently emerged, e.g., using curvature in cylindrical nanowires as a geometrical mechanism to nucleate Bloch points with well-defined polarity.^30^

Here, we extend the paradigm of 3D curvature for deterministic Bloch point nucleation in chiral nanowires with double-helix geometry, where the intrinsic chirality of the helical wires provides direct control over key internal degrees of freedom of circulating Bloch points, namely, their circulation, helicity, and polarity. By interfacing left- and right-handed helices, we introduce a chirality interface where the geometric chirality reverses, which, in turn, acts as a robust nucleation site for Bloch point domain walls. The deliberate curvature introduced at the chirality interface further enables the initialization of either head-to-head or tail-to-tail Bloch point walls under applied magnetic fields. Careful tailoring of the nanostructure thus establishes a direct correspondence between the geometrical topology (i.e., reversal of geometric chirality) and magnetic topology (i.e., emergence of Bloch point singularities). Importantly, the chirality of the nanowires imprints a well-defined circulation on the Bloch points, in contrast to high-symmetry cylindrical nanowires or nanospheres,^16,17,28,30^ where left-handed and right-handed circulations are energetically degenerate.

Our approach is conceptually analogous to previous theoretical proposals of interfacing films with opposite signs of the Dzyaloshinskii–Moriya interaction (DMI) to stabilize circulating Bloch points.^10,44,45^ However, engineering such finely balanced thin-film layers can be challenging, as both the sign and magnitude of the DMI will need to be tuned with high accuracy. Furthermore, thin-film systems currently lack practicable protocols to initialize and address Bloch points in a reliable manner and instead rely on intrinsic defects as nucleation sites.^29,46,47^

In contrast, by experimentally controlling the geometric—and thus magnetic—chirality in a tailored 3D geometry made from a single material, we here demonstrate full control over the spin texture of circulating Bloch points. We note that the transverse initialization fields used in the main experiments are relatively high, of the order of 1 T, and were chosen to ensure robust and reproducible Bloch-point nucleation in proof-of-principle experiments. An alternative field protocol using lower field magnitudes of the order of 100 mT, more suitable for in situ studies, is discussed in Supporting Information Section S5.

This geometrical approach to nucleate Bloch points with tailored spin textures provides a powerful platform for experimental exploration of Bloch point physics, including their field-driven or spin-transfer-torque-driven motion, their high-frequency response,^14,19,24,45^ and nonreciprocal interactions with current-generated Oersted fields.^11,16,23,26^ Importantly, the deterministic relation between Bloch-point polarity and circulation achieved here enables direct coupling between the circulating Bloch-point spin state and current-induced Oersted fields circulating around the nanowire, enabling selective actuation depending on the internal state and current direction. Geometric selection and control of Bloch points therefore hold strong potential for future Bloch-point-based spintronic devices, including racetrack memory and logic architectures.^4,25,44^

## 5 Methods

### 5.1 Sample Growth

Magnetic Co double helix structures were grown on precut Cu TEM grids using focused electron beam induced deposition (FEBID) with a Co_2_(CO)_8_ precursor, using a Thermo Fisher HELIOS-600 Dual beam SEM+FIB. The geometries were defined as 3D stl files and transformed to stream files, which are readable to the microscope, using the open-access code f3ast.^35,48^ Accelerating voltages of 5 kV and a beam current of 43 pA at a chamber pressure of 3 × 10^–6^ mbar at growth were used, leading to initial growth rates of GR_0_ = 40–70 nm s^–1^. A variable dwell time increasing with growth height was used to account for thermal effects during the nanowire growth.^36,48^

The individual helix strands have a diameter of 85 nm, while the helix is characterized by a periodicity of 250 nm and a nominal gyration radius of 34 nm (corresponding to a strand overlap of 18 nm). These parameters are similar to those of previous works.^31,36^ Each chiral section is about 750 nm long, with the left-handed segment on the bottom standing on a short straight pedestal.

### 5.2 Magnetic Soft X-ray Nanotomography

Magnetic vector tomography was performed at the MISTRAL beamline of the ALBA synchrotron, with X-rays tuned to the Co *L*_2_ absorption edge at a nominal energy of 794.8 eV.

#### 5.2.1 Bloch Point Initialization

The Bloch-point state was initialized ex situ by applying a magnetic field along the *x* direction of the nanostructures of about 1.2 T using an electromagnet. After the field was reduced to zero, the sample was transferred to the beamline X-ray transmission microscope.

#### 5.2.2 Data Acquisition

The lateral resolution of the microscope (≈30 nm) is largely determined by the objective Fresnel zone plate lens used.^49^ A magnification of ×1300 was employed, leading to an image pixel size of 10 nm. To reconstruct the 3D magnetization **m**(**r**), images from two tilt series were recorded: first, for rotations around the main axis of the nanowire from −65° to +64° in 1.5° steps, and second, for tilts around the *y*-axis from −33° to +19° in 1° steps. At each angle, measurements were taken with left- and right-handed polarization, *C*_*L*_ and *C*_*R*_, respectively. Flat field images with the sample removed from the imaging path were measured every hour for both polarizations; these allow correction for inhomogeneous illumination and quantitative determination of sample transmittance before calculating the structural and XMCD contrast (see Supporting Information Section 1.1 for image processing details). For each projection angle and X-ray polarization, 40 images (with a total exposure time of 120 s) were taken and subsequently averaged to increase the signal-to-noise ratio.

#### 5.2.3 Structural and Magnetic Contrast

The postprocessing of the data to compute the X-ray transmittance and correct for image shifts in order to obtain averaged transmission images with left- and right-handed X-ray polarization, *T*_*L*_ and *T*_*R*_, is described in Supporting Information Section S1.1. The relative shift between features in *T*_*L*_ and *T*_*R*_ is obtained from image registration, with $T_{R}^{\prime }$ being the shifted *C*_*R*_ image. From this, the structural contrast (optical density) and XMCD contrast corresponds to the average and difference of the natural logarithm of the two images, respectively:

3 $$\mathrm{OD}=\frac{1}{2}\log\left(T_{L}T_{R}^{\prime }\right)$$

4 $$\mathrm{XMCD}=\frac{1}{2}\log\frac{T_{R}^{\prime }}{T_{L}}$$

#### 5.2.4 Tomographic Reconstruction

The aligned structural contrast and XMCD tilt series were used as input to perform the structural and magnetic vector reconstructions using a Simultaneous Iterative Reconstruction Technique (SIRT) evolved from the one described in ref 40. Further details of the reconstruction process are explained in Supporting Information Section S1.2. The reconstructions of the scalar electronic density and vector magnetization **m**(**r**) were obtained with a voxel size of (10 nm)^3^, result of the pixel size defined by the magnification in the microscope. The reconstruction resolution is estimated to be ≈50 nm, similar to previous experiments.^21^ 3D data representation and streamline computation were done with paraview.

### 5.3 Transmission Electron Microscopy

Transmission electron microscopy was performed using an FEI Titan^3^ 80–300 TEM instrument (Thermo Fisher Scientific, US) operated in Lorentz mode at an acceleration voltage of 300 kV.

#### 5.3.1 Off-Axis Electron Holography

To map the remanent magnetic configuration of the helical nanowires after Bloch point nucleation, off-axis electron holography was employed. In this technique, an electron wave passing through a magnetic specimen acquires an Aharonov-Bohm phase shift with respect to a vacuum reference. This phase shift contains contributions from both the electrostatic potential and the magnetic vector potential of the sample. Holograms were recorded using an electrostatic Möllenstedt biprism biased at +250 V, which yields a fringe spacing of 1.2 nm. The holographic images were captured with a Gatan OneView CMOS camera, providing a fringe contrast of approximately 10% in vacuum. In order to disentangle the electrostatic and magnetic contributions to the measured phase shift, each hologram was acquired twice: first with the specimen in its original orientation and then after flipping the sample upside-down. The sum of the two phase reconstructions yields twice the electrostatic phase, whereas their difference provides twice the magnetic phase, enabling a quantitative separation of the two components. In most cases, the holograms were recorded at an angle of −40° (see Figure 5b,c), while the nanowire kink points out-of-plane at an angle of 0°.

#### 5.3.2 Bloch Point Initialization

To nucleate a Bloch point in the double-helical Co nanowires, an external saturation field of about 0.3 T was applied by exciting the objective lens (which is normally switched off in Lorentz mode imaging). The nanowire was first aligned with the TEM goniometer so that the external field direction lies in the plane of the kink orthogonal to the wire axis (i.e., *H*_ini_∥*x*). This configuration ensures that the magnetization is driven into a saturated state, transverse to the wire. After saturation, the field was gradually decreased to zero. The specimen was then rotated back to its original orientation, and the remaining magnetic configuration was recorded at zero applied field.

#### 5.3.3 Electron Holographic Tomography

Two tilt series of off-axis electron holograms were acquired over a tilt range of −71° to +69° in 2° increments, aligning the tilt axis along the nanowire axis. After acquiring the first series, the specimen was flipped upside-down outside the microscope, and a second series was recorded under identical imaging conditions, providing the opposite magnetic polarity necessary for separating electric and magnetic phase shifts and hence fields. The holograms were reconstructed by Fourier filtering to yield two tilt series of phase images. The latter were subjected to the alignment protocol described by Wolf et al.,^37^ which included, e.g., correcting for image displacements via cross-correlation and center-of-mass approach within a tilt series, registering the tilt axis, and correcting for displacements between the original and flipped series. From the added and subtracted phase maps, the electrostatic phase (∝ *V*(*x*,*y*,*z*)) and the magnetic phase (∝ *B*_*z*_(*x*,*y*,*z*)) were extracted, giving access to the three-dimensional electrostatic potential *V*(*x*,*y*,*z*) (structural information) and the magnetic-induction component parallel to the tilt axis, *B*_*z*_(*x*,*y*,*z*). For the 3D reconstruction, the aligned phase stacks were fed into the weighted simultaneous iterative reconstruction technique (W-SIRT)^50^ to obtain quantitative tomograms of both *V*(*x*,*y*,*z*) and *B*_*z*_(*x*,*y*,*z*). More details about the TEM measurements and data analysis are provided in Supporting Information Section S4.

#### 5.3.4 Relation between Electron and X-ray Reconstruction of the Magnetization

The magnetic contrast mechanisms used in this work are fundamentally different, with electron holography having nonlocal sensitivity to the magnetic induction **B**, whereas XMCD is proportional to the local magnetization *m*∥*k*. Regardless, both methods ultimately probe the same physical quantity, since XMCD is sensitive only to the solenoidal (divergence-free) part of the magnetization vector field, which is equivalent to **B**.^46^ Our measurements thus provide direct access to the three-dimensional spin texture surrounding the Bloch point, with the singular core remaining below the experimental resolution.

### 5.4 Micromagnetic Simulations

Finite-element simulations were run using custom code magnum.pi.^51^ The double-helix geometry shown in Figure 1e was defined using Python-based cadquery, and then segmented into a triangulated mesh using open-source software gmsh and salome. The meshed-up geometry had a median node spacing of approximately 6 nm, comparable to the exchange length of 5.7 nm, which is sufficient to capture the surrounding micromagnetic spin texture. However, continuum micromagnetics cannot resolve the Bloch-point core itself, where the fixed-length magnetization approximation breaks down. A quantitative description of the singular core would require atomistic or hybrid atomistic–micromagnetic modeling and is beyond the scope of the present work.

Magnetic material parameters were chosen as *M*_sat_ = 700 kA m^–1^, *A*_ex_ = 10 pJ m^–1^, and *K* = 0, similar values to previous works with FEBID cobalt.^41^ α = 1 was used to reduce computation times since we focus here on equilibrium states. No intrinsic DMI (i.e., *D* = 0) was assumed, and the chiral response purely originates from the imprinted shape of the double-helix geometry.

To obtain the remanent spin state of a Bloch point, the initial magnetic state was set to be saturated along the *x* direction, and then a magnetic field *H*_*x*_ parallel to the initial magnetization was reduced from ±180 mT to zero in 0.5 mT steps. The LLG time evolution was performed in discrete time steps of 10 ps, with at least ten steps at each field to relax toward an equilibrium spin state.

Subsequent analysis of the simulated magnetic state and its topological properties has been performed using the Python-based package pyvista. Derived quantities from the magnetization **m**(**r**) include the magnetostatic volume charge, given by the divergence ρ_*m*_ = ∇·**m**(**r**),^52^ and the emergent vorticity field **Ω**(**r**)^41^

5 $$\Omega_{i}\left(r\right)=\frac{1}{8\pi }\epsilon_{ijk}\epsilon_{rst}m_{r}\partial_{j}m_{s}\partial_{k}m_{t}$$

where ϵ_*ijk*_ and ϵ_*rst*_ denote the antisymmetric Levi-Civita tensor, and indices *i*, *j*, *k*, *r*, *s*, *t* = {*x*, *y*, *z*}.^53^

## Supplementary Material









## Data Availability

Data and codes can be found at the Zenodo repository at https://doi.org/10.5281/zenodo.23234691.

## Notes

Volumetric render of the electrostatic potential (MP4)
Alternative view on the electrostatic potential of the nanostructure close to the chirality interface (MP4)
3D view of magnetic induction within the nanostructure (MP4)
Information regarding XMCD and TEM methods (Sections S1 and S4, respectively), a model for the deterministic Bloch point displacement (Section S2), determination of the Bloch point helicity angle (Section S3), and an alternative Bloch-point initialization protocol (Section S5) (PDF)
