# Supplementary material for: Generation of Bloch Points with Controlled Spin Texture Using Geometrical Boundary Conditions
Source: ACS Nano. 2026 Oct 13;20(42):28359–69. doi: 10.1021/acsnano.6c09658 (PMC13718616; doi:10.1021/acsnano.6c09658)
Supplement: Supplementary file 1 [file nn6c09658_si_004.pdf]

Generation of Bloch Points with Controlled Spin Texture Using Geometrical Boundary Conditions Supporting Information

Naëmi Leo[*,1,2], Daniel Wolf[3], Alicia Estela Herguedas-Alonso[4,5,6], Oleksandr Zaiets[3,7], Jakub Jurczyk[2], Takeaki Gokita[2], John Fullerton[8], Dedalo Sanz-Hernandez[9], Claire Donnelly[10], Andrea Sorrentino[6], Eva Pereiro[6], Lucia Aballe[6], Peter Fischer[11,12], Rachid Belkhou[13], Claas Abert[14], Dieter Suess[14], Axel Lubk[3,7], Aurelio Hierro-Rodriguez[4,5], and Amalio Fernández-Pacheco[†,2]

[1]Department of Physics, Loughborough University, LE11 3TU Loughborough, United Kingdom
[2]Institute of Applied Physics, TU Wien, Vienna 1040, Austria
[3]Leibniz Institute for Solid State and Materials Research Dresden, 01069, Dresden, Germany
[4]Departamento de Física, Universidad de Oviedo, 33007, Oviedo, Spain
[5]CINN (CSIC—Universidad de Oviedo), 33940, El Entrego, Spain
[6]ALBA Synchrotron Light Source, 08290 Cerdanyola del Valles, Barcelona, Spain
[7]Institute of Solid State and Materials Physics, Technische Universität Dresden, 01069, Dresden, Germany
[8]Materials Science Division, Argonne National Laboratory, Lemont, IL, 60439 USA
[9]Laboratoire Albert Fert, CNRS, Thales, Palaiseau, 91767, France
[10]Max Planck Institute for Chemical Physics of Solids, 01187, Dresden, Germany
[11]Materials Sciences Division, Lawrence Berkeley National Laboratory, Berkeley, CA 94720, USA
[12]Physics Department, University of California Santa Cruz, Santa Cruz, CA 94056, USA
[13]Synchrotron SOLEIL, Saint-Aubin 91190, France
[14]Faculty of Physics, University of Vienna, Vienna 1090, Austria

[*]n.leo@lboro.ac.uk, [†]amalio.fernandez-pacheco@tuwien.ac.at

S1 XMCD Tomography

S1.1 X-ray Image Post-Processing

As described in Methods Sec. 5.2, for each sample rotation and tilt angle x-ray images $I_{p,j}$, with p denoting the xray polarisation, i.e., $p = C_L$ or $p = C_R$, respectively, and $j = 1 \ldots 40$ the image index of equivalent images taken at a given setting to improve the signal-to-noise ratio. Additionally, regular flat field exposures $F_{i,j}$ with the sample removed from the light path were taken to correct for an inhomogeneous background illumination and to compute the transmittance.

In the following, we use the symbol $\langle\cdot\rangle$ to denote the median, calculated using the function `numpy.nanmedian`. In particular, $\langle\cdot\rangle_j$ denotes the pixel-by-pixel median over a stack of 40 images. We found that this function produced better statistics compared to the normal average (i.e., mean).

Calculation of transmittance. The background illumination in full-field transmission x-ray microscopy is not homogeneous because of the shaking of the capillary condenser; the background also shows slight fluctuations in overall intensity over time. To correct for these spatial and temporal

background fluctuations, for a given polarization p, first we obtain the corrected intensity, respectively transmittance, $T_{p,j}^{\mathrm{corr}}$ in which the background signal is normalized to 1 via the following equation:

$$T_{p,j}^{\mathrm{corr}} = \frac{I_{p,j}}{f_p \left\langle F_{p,j} \right\rangle_j} \,. \tag{S1}$$

The normalization factor f_p can be calculated via the ratio of the image and the flat field at the same polarization p, where the "mask" selects all pixels that constitute the background, i.e., which lie outside of the structures where the transmittance is unity (here, the mask was implemented using an intensity threshold function):

$$f_p = \left\langle \left. \frac{I_{p,j}}{\left\langle F_{p,j} \right\rangle_j} \right|_{\mathrm{mask}} \right\rangle \,. \tag{S2}$$

Image registration. Relative drifts between $T_{p,j}^{\mathrm{corr}}$ images at $j = 0$ and j were obtained using a combination of an edge-filter (using `skimage.feature.canny`) and a Fourier-based cross-correlation algorithm with sub-pixel precision `skimage.registration.phase_cross_correlation` [1]. The average T_p (i.e., T_L and T_R for both polarisations) with the background normalized and images shifted to a common feature $T_{p,j}^{\mathrm{corr}\prime}$ can then be calculated as

$$T_p = \left\langle T_{p,j}^{\mathrm{corr}\prime} \right\rangle_j \tag{S3}$$

Structural and magnetic xray contrast. Similar to the previous step, the relative shift between features in T_L and T_R are obtained from image registration, with T_R' being the shifted C_R image. From this, the optical density (i.e., the electronic or structural contrast) and XMCD contrast corresponds to the average and difference of the natural logarithm of the two images, respectively:

$$\mathrm{OD} = \frac{1}{2} \log \left(T_L \, T_R' \right) , \tag{S4}$$

$$\mathrm{XMCD} = \frac{1}{2} \log \frac{T_R'}{T_L} \,. \tag{S5}$$

In this notation, a positive XMCD contrast indicates magnetic moments pointing opposite to the x-ray k vector, i.e., towards the viewer.

S1.2 Tomographic Reconstruction

Image series alignment and masking. All images in the two series, i.e., rotation around the z axis of the nanostructure and tilts around the y' axis (which is roughly parallel to x, but not necessarily perfectly perpendicular to z), were aligned manually to each other. Using the structural (charge) contrast, masks were created for each charge and XMCD image, to limit the input data for the reconstruction.

Tomographic reconstruction. The workflow of the XMCD tomographic reconstruction is illustrated in Fig. S1. The aligned structural contrast and XMCD tilt series were used as input to perform the structural and magnetic vector reconstructions using a Simultaneous Iterative Reconstruction Technique (SIRT) evolved from the one described in Ref. [2]. For both charge and magnetic reconstructions 20 SIRT iterations were employed.

Reconstruction of charge density. First, the scalar density was reconstructed from the charge contrast data allowing to obtain the sample geometry. The two experimental rotation axes may neither be fully aligned with the coordinate system of the nanostructure, and, more importantly, not necessarily perfectly perpendicular to each other. Therefore, a geometric transformation is found that maps the reconstructed charge density of each series onto the other.

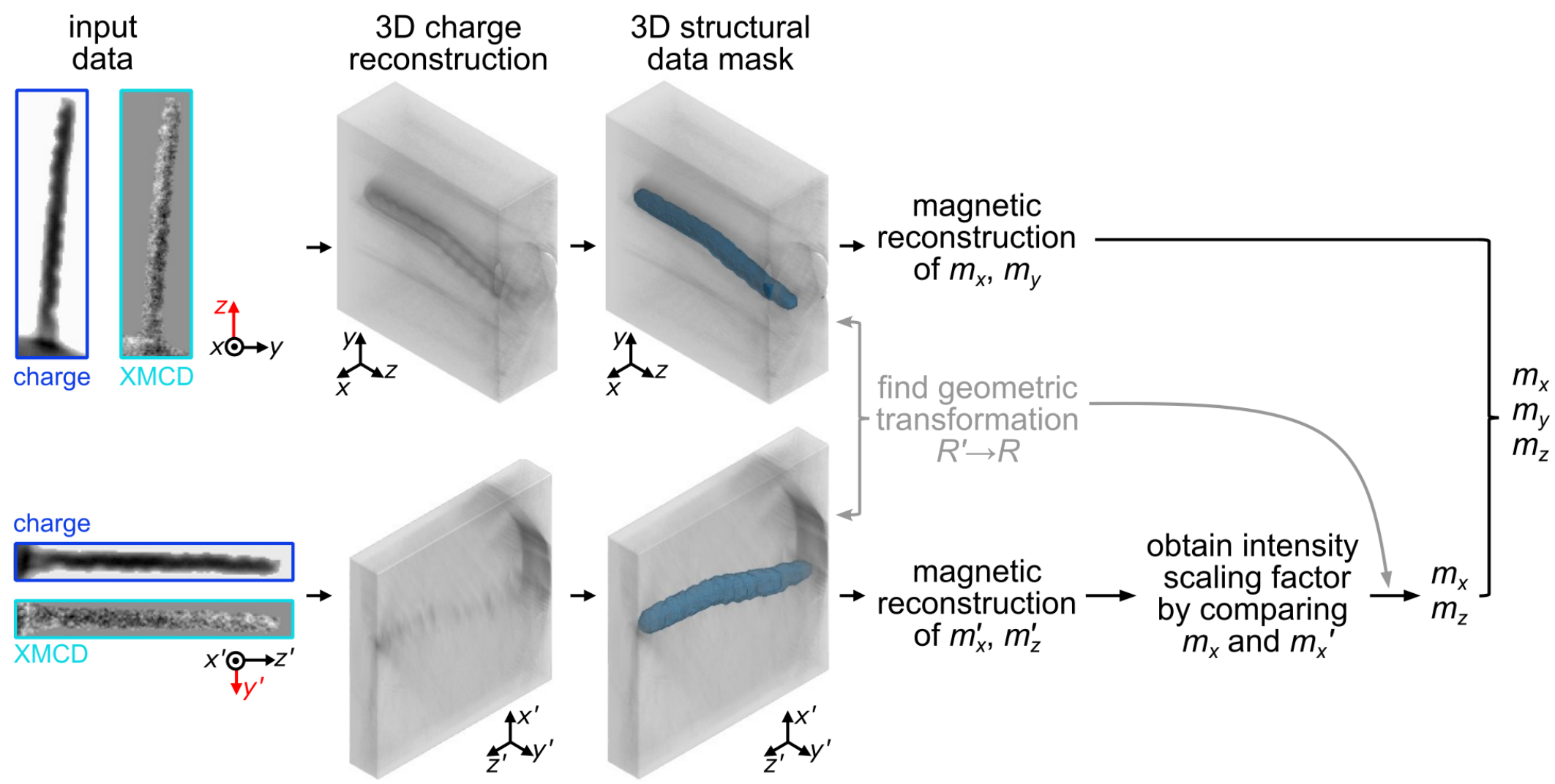

Figure S1: **XMCD tomography reconstruction workflow.** See Sec. S1.2 for details.

Reconstruction of 3D magnetization. The magnetic information is computed from the XMCD images constraining the reconstruction volume to the geometry obtained from the charge density 3D reconstruction. As XMCD is only sensitive to the magnetization component parallel to the k vector, i.e., $m_k \parallel k$, for each series two components of the solenoidal part of the three-dimensional magnetization can be reconstructed. These are m_x and m_y for the rotation around z, and m'_x and m'_z for the rotation around the y' axis. As the values of m_x and m'_x should be the same, these are compared to find a global conversion factor which allows to put in the same scale m'_z from the second tilt series. Finally, using the previously determined geometric transformation $R' \to R$, the magnetization components in the frame of reference of the first tilt series can be computed obtaining m_x, m_y, and m_z.

S2 Curvature-Driven Bloch Point Displacement

As shown previously [3], Bloch point domain walls are energetically unfavorable in curved nanowire sections. The field initialization procedure illustrated in Fig. 1 implies nucleation of a Bloch point at or close to the chirality interface placed at the kink in the helical nanowire. Therefore, it can be expected that the Bloch point will displace away from the kinked region upon reducing the magnetic field to zero.

The two Bloch points selected by the geometry of the helical nanowire shown in Figs. 1(f,g) – clockwise head-to-head and counter-clockwise tail-to-tail – can be transformed into each other by time reversal symmetry, and thus have the same energy at remanence. Furthermore, the Bloch points are achiral and have net zero magnetization. This implies that there are no specific symmetry breaking terms to the static energy of the remanent state that explain how the magnetic texture is driven out of the high-curvature region of the nanowire.

Arguments for Bloch point displacement via the LLG damping term. Instead, we find that during the initialization procedure described in Fig. 1, the coupling between the polarity and its emergent circulation gives rise to an additional torque in a curved wire. As a result the Bloch point is expected to displace from the central chirality interface along a well-defined direction.

In the double-helix geometry shown in S2(a), the Bloch point domain wall experiences a torque

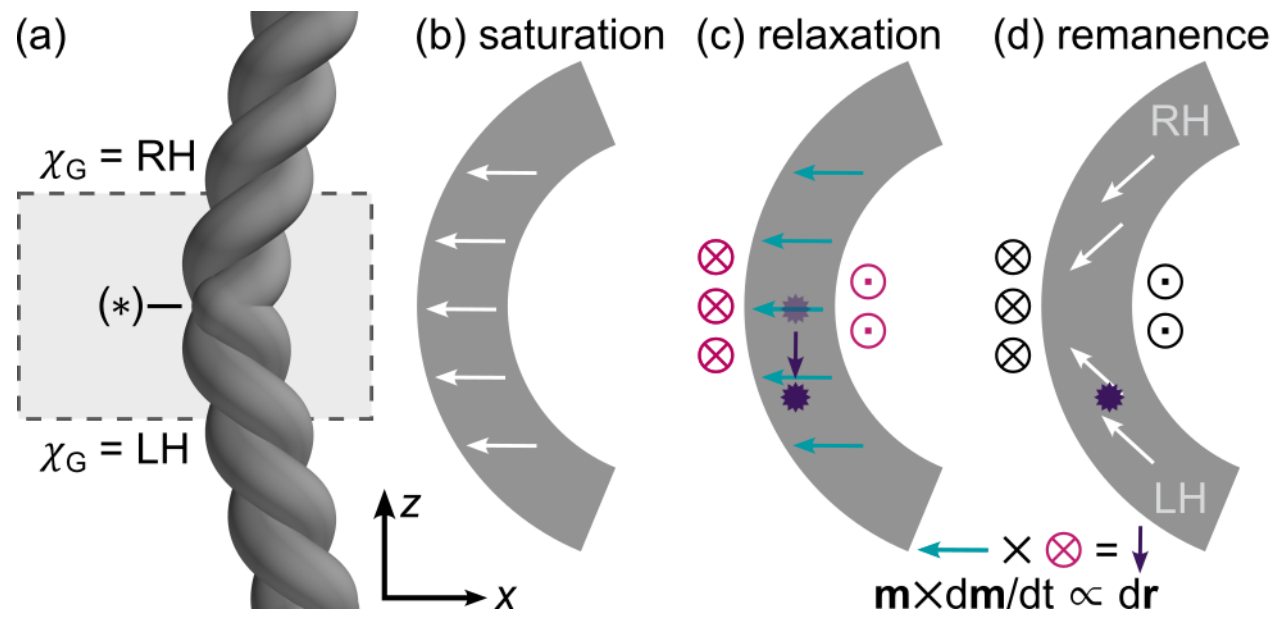

Figure S2: **Deterministic shift of a Bloch point domain wall**, in a curved nanowire, as a result of the damping term of the Landau-Lifschitz-Gilbert equation. (a) Double-helix geometry with a chirality interface at (∗). For this explanation, the kink is simplified to a segment of constant curvature. (b) Field-set saturated state with $\mathbf{m} \parallel x$. (c) During magnetic relaxation, the emergence of circulation at the curved segment leads to an imbalance of $\frac{dm_y}{dt}$ contributions, leading to a net torque $\tau_z \propto m_x \times \frac{dm_y}{dt}$ acting on the Bloch point domain wall. (d) As the combination of the initializing field and the geometric control imposes a deterministic circulation to emerge, the Bloch point displacement is deterministic towards the left-handed (right-handed) segment for a head-to-head (tail-to-tail) configuration.

driven by the (second) damping term to the Landau-Lifschitz-Gilbert (LLG) equation:

$$\frac{\mathrm{d}\mathbf{m}}{\mathrm{d}t} = -\gamma \mathbf{m} \times \mathbf{h}_{\text{eff}} + \alpha \mathbf{m} \times \frac{\mathrm{d}\mathbf{m}}{\mathrm{d}t} \,. \tag{S6}$$

This additional torque can be intuitively understood using the schematics shown in Fig. S2(b-d): For a high field $\mathbf{H} \parallel x$, Fig. S2(b), an initial saturated state with all moments $\mathbf{m} \parallel x$ is achieved. Upon reducing the field to zero, this configuration will relax towards the remanent state shown in Fig. S2(d), leading to the stabilization of a Bloch-point domain wall. The sign of the initial H_x field implies a head-to-head final configuration is selected in the sketched case. Importantly, the geometric design of the helical wires, i.e., the order of left-handed and right-handed wire segments, imposes a specific circulation (here clockwise) that emerges during the relaxation process. The evolution during the relaxation process, Fig. S2(c), is therefore governed by the shape anisotropy, rotating initial $\mathbf{m} \parallel x$ moments towards the y direction (to establish the circulation) as well as $\pm z$ (to set the polarity). As the structure shows a kink (simplified in Fig. S2 to a segment of constant curvature), and the nanowire diameter is finite, upon reduction of the magnetic field the m_y component shows a transient evolution that is asymmetric, with a curvature-induced net contribution

$$\left.\frac{|\mathrm{d}m_y|}{\mathrm{d}t}\right|_{\text{outer}} > \left.\frac{|\mathrm{d}m_y|}{\mathrm{d}t}\right|_{\text{inner}} , \tag{S7}$$

leading to a deterministic torque $\tau_z \propto m_x \times \frac{\mathrm{d}m_y}{\mathrm{d}t}$ that displaces via $\mathrm{d}\mathbf{r}_z \propto \tau_z$ the Bloch point towards the (bottom) left-handed segment for a head-to-head configuration.

Displacement direction. For a tail-to-tail configuration, both the sign of the initial m_x component reverses as well as the final circulation, leading to an opposite displacement to the right-handed segment. Upon reversal of the geometric order of left-handed and right-handed segments, the opposite magnetic circulation is stabilized with applied H_x field, thus also inverting the direction of the displacing torque, resulting in a displacement towards the section of the same geometrical chirality as before. In conclusion, regardless of the order of the left- and right-handed nanowires, a head-to-head (tail-to-tail) Bloch point will displace towards the left-handed (right-handed) segment.

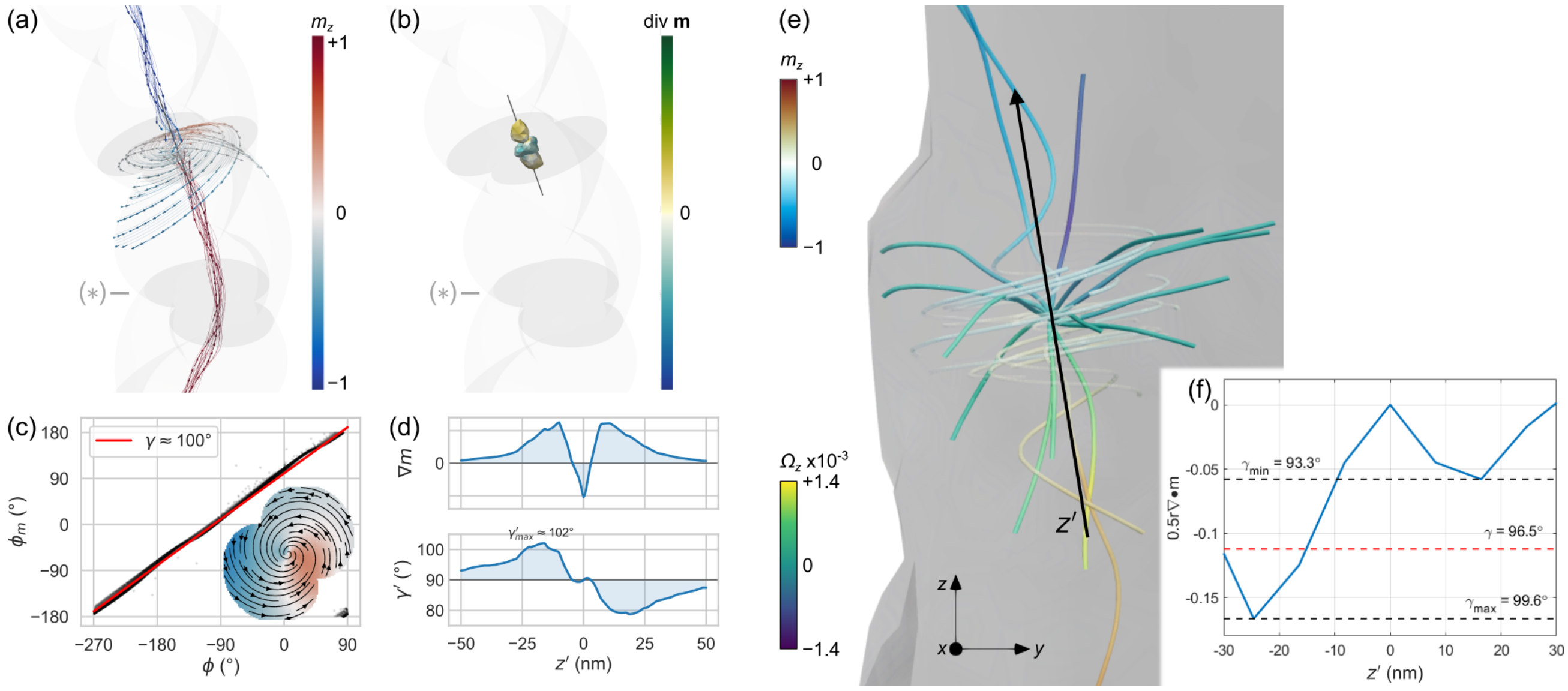

Figure S3: **Determination of helicity angle γ.** (a) Streamline magnetization plot of a head-to-head Bloch point. Its equatorial plane and main axis are indicated, as well as the chirality interface (∗). (b) Double-lobed iso-surfaces of the magnetostatic charge $\nabla\cdot\mathbf{m}$ indicate the Bloch points' hyperbolic character. (c) Plot of ϕ_m against ϕ within the equatorial plane. The inset shows the spin configuration within the equatorial plane, demonstrating the inwards tilt of the circulating magnetization. (d) Top: Magnetostatic charge $\nabla\cdot\mathbf{m}$ along the z' axis of the Bloch point. Bottom: Estimation of the helicity angle via Eq. (S8). (e,f) Determination of γ from experimental XMCD tomography. (e) Stream tracer of magnetisation $\mathbf{m}$ and magnetic vorticity $\mathbf{\Omega}$ of the experimental configuration. (f) The variation of Eq. (S8) along the main axis z' of the Bloch point [marked black in (e)], indicates a maximum angle of $\gamma \approx 99.6°$, in agreement with simulations.

Bloch points in achiral wires. The relaxation-driven displacement Δz of a Bloch point from a high-symmetry point within a curved region conversely allows to *indirectly* indirectly infer the sense of circulation of a Bloch point initialized in *achiral* curved nanowires [4]. Defining the circulation sign as $\sigma(\gamma) = +1$ for counter-clockwise and -1 for clockwise circulation when viewed from $+z$ downwards, the displacement obeys $\sigma(\Delta z) \propto \sigma(\gamma)$. Within this LLG-based picture, a deterministic shift of a Bloch point domain wall relative to a curved section is therefore expected. In experimental systems, however, pinning at local inhomogeneities or small misalignments of the initialization field may dominate over this geometry-induced dynamic displacement. Based on TEM and XMCD imaging data, we argue in the main manuscript that this is indeed the case for our nanostructures, as the observed displacement does not always correspond to the predicted from the damping term of the LLG equation.

S3 Determination of the Helicity Angle γ

Figure S3(a) shows the spin structure $\mathbf{m}(\mathbf{r})$ in the vicinity of the Bloch point, where the two opposing vortex cores meet. The circulating moments indicate the formation of a Skyrmion-tube-like configuration [5] in the segment closer to the chirality interface at (∗), as the core axial magnetization (in red) opposes the m_z component of the outer spins (in blue).

From the spin state we determined the orientation of the main axis z' of the Bloch point, which is about 3° inclined to the main axis of the helical section tilted at θ_{tilt}=15°. The Bloch point axis as well as the perpendicular equatorial plane are indicated in Figs. S3(a,b). Figure S3(b) shows the iso-surfaces of the magnetostatic charge distribution $\nabla \cdot \mathbf{m}(\mathbf{r})$, showing a double-lobed configuration around the Bloch point, indicating its hyperbolic character [6].

To determine the value of the helicity angle γ we used two different approaches.

First, we directly consider the spin configuration in the equatorial plane of the Bloch point, which is shown as inset in Fig. S3(c). Using Eq. (2), one can extract γ from relating the spin angle ϕ_m to the real-space polar angle ϕ. To determined ϕ from the (x, y) coordinates, the center of mass of the simulated exchange energy $\propto \Sigma_{i,j}(\partial_j m_i)^2$ was used, as this point corresponds the central position of the Bloch point. The resulting $\phi_m(\phi)$ graph is shown in Fig. S3(c), and from linear regression we obtained value of $\gamma_{\text{sim}} \approx 100°$. The fact that $\gamma > 90°$ indicates an inwards tilt to the circulation, as is evident in the inset where the in-plane moments slightly spiral inwards.

Second, the helicity angle γ can be obtained by an approximate formula proposed in [6], involving the magnetostatic charge, respective divergence $\nabla \cdot \mathbf{m}$, along the main axis z':

$$\cos(\gamma') = \frac{z'}{2} \nabla \cdot \mathbf{m} \tag{S8}$$

Fig. S3(d) shows the evolution of $\nabla \cdot \mathbf{m}$ along z' on the top, and the term from Eq. (S8) on the bottom. A maximum value of $\gamma'_{\text{sim}} \approx 102°$ is observed very close ($\approx 20\,\text{nm}$) to the Bloch point, matching the value of $\gamma_{\text{sim}} \approx 100°$ from $\phi_m(\phi)$ above.

We used the second method to perform an equivalent analysis for the reconstructed XMCD tomography data $\mathbf{m}(\mathbf{r})$, as shown in Figs. S3(e,f); which returned an experimental value of $\gamma'_{\text{exp}} \approx 99.6°$, in agreement with simulations.

S4 2D and 3D Transmission Electron Microscopy

Electron holography. Electron holograms at the position shown in Fig. S4(a) were recorded and reconstructed to obtain phase images covering 750 nm of the entire nanowire (see Fig. S4(a)) around its chirality interface. The phase shift φ in the object plane (x, y) is given by

$$\varphi(x, y) = \int_{-\infty}^{+\infty} \left(\frac{e}{\hbar v} V(x, y, z) - \frac{e}{\hbar} A_z(x, y, z) \right) \mathrm{d}z. \tag{S9}$$

Here, v represents the electron velocity, $V(x, y, z)$ the 3D electrostatic potential, e the elementary charge, $\hbar$ the reduced Planck constant, and $A_z(x, y, z)$ the 3D z-component of the magnetic vector potential. We can refer to the first term as electric phase shift φ_{el} and the second term as magnetic phase shift φ_{mag}.

By converting the second integral to the magnetic flux enclosed between interfering paths of object and reference wave, the directional spatial derivatives read

$$\begin{pmatrix} \partial_x \varphi_{\text{mag}} \\ \partial_y \varphi_{\text{mag}} \end{pmatrix} = \frac{e}{\hbar} \int_{-\infty}^{\infty} \begin{pmatrix} B_y(\mathbf{r}) \\ -B_x(\mathbf{r}) \end{pmatrix} \mathrm{d}z\,; \tag{S10}$$

hence, they are proportional to the projected in-plane components of the magnetic induction $\mathbf{B}$.

To quantify the electrostatic mean inner potential and the magnetic induction, the electric φ_{el} and magnetic phase φ_{mag} shifts must to be separated. This is accomplished by first reversing the

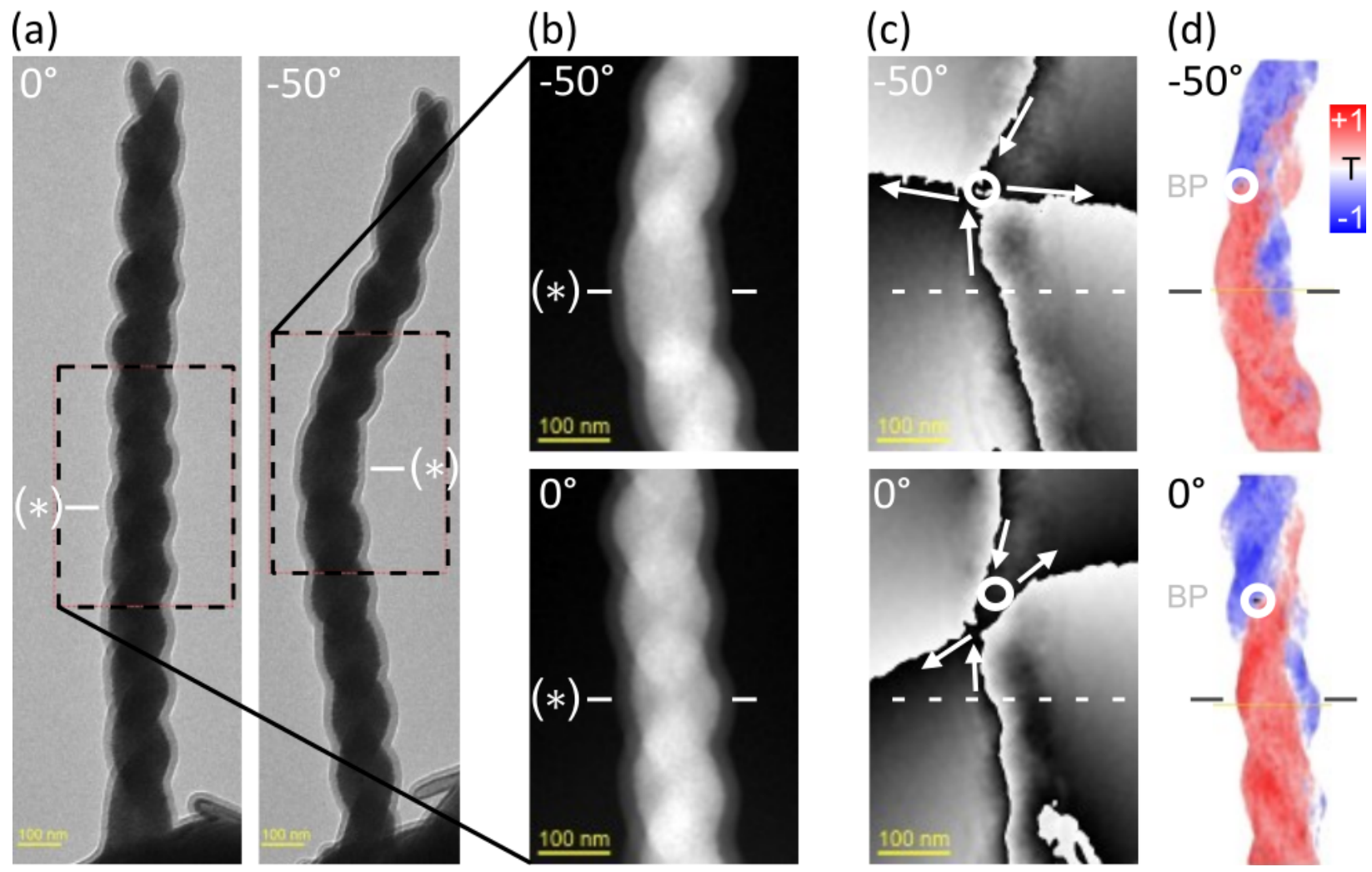

Figure S4: **Electron holographic tomography.** (a) Bright-field transmission electron microscopy images of the double-helix cobalt nanowire. The position of the chirality interface is marked with (∗), and the chosen field of view for tomographic reconstruction around the kink highlighted with a dashed box. (b) Electrostatic and (c) magnetic phase images for different tilt angles. In (c), the phase wraps modulo two π, amplifying equi-phase lines, illustrate the projected B-field lines (white arrows), which are consistent with the internal B_z component visualized as red-blue volume rendering in (d). The inferred Bloch point position is indicated by white circles.

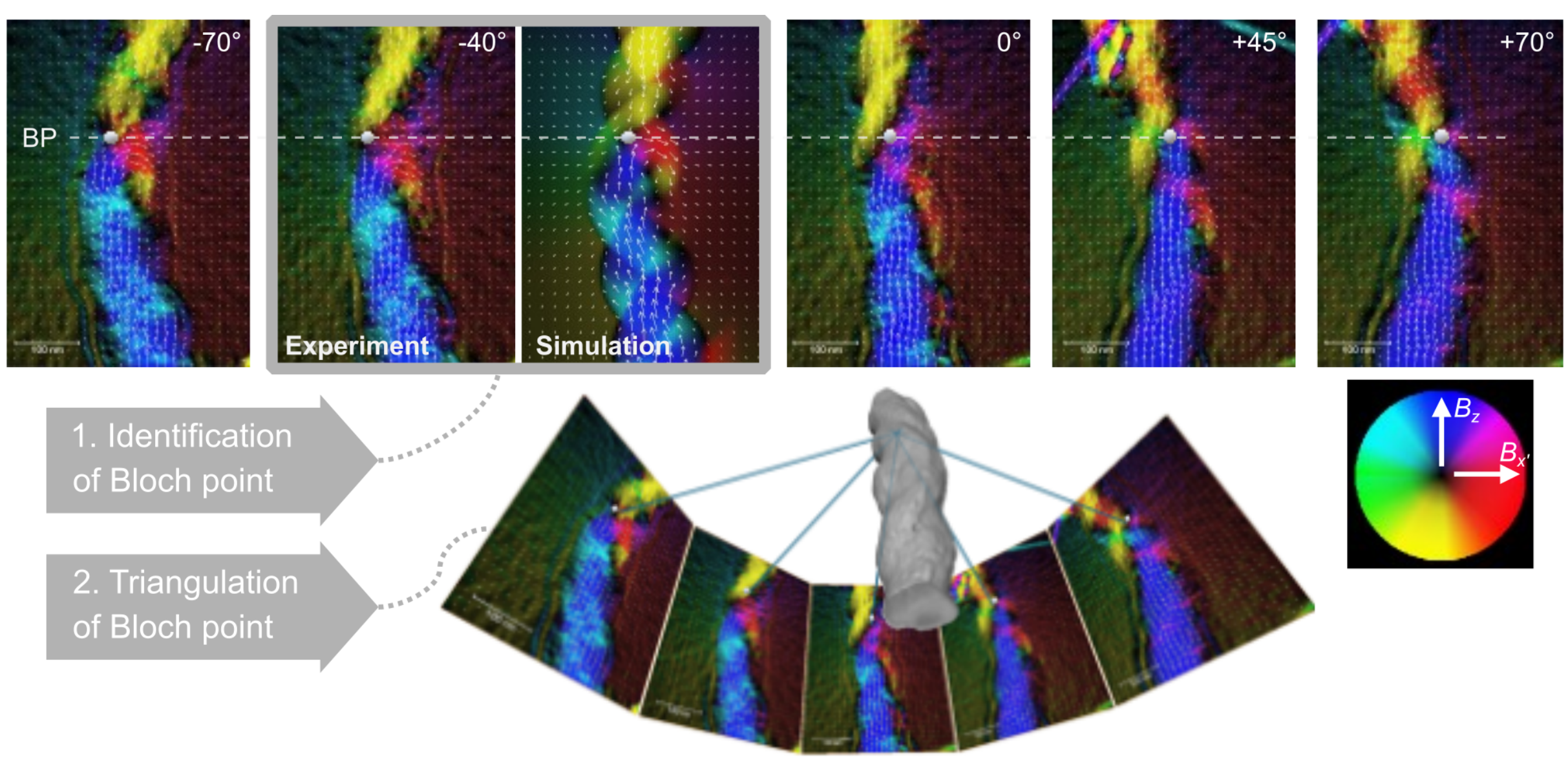

Figure S5: **Determination of the Bloch point position** from TEM holography. First, comparison with simulation results allows to identify the position of the Bloch point within a specific 2D induction phase map projection. Second, combining the positions from three or more projections allows to triangulate the Bloch point position in 3D.

magnetic phase shift (e.g., by flipping the sample upside down), and second, reconstructing the phase image of the same object again [7]. Then, the sum (difference) of phase images before and after flipping yields twice the electrostatic (magnetic) phase shift φ_{el} (φ_{mag}). Electric and magnetic phase images within the field of view for 0° and −50° are shown in Fig. S4(b,c). As indicated by the white arrows, the magnetic field lines seem to converge to the Bloch point position (white circle).

Electron holographic tomography. To reconstruct the three-dimensional data presented in Fig. 4 after the Bloch point initialization, a field of view around the central part of the double-helical Co nanowire was chosen for a tomographic measurement series. This region, as outlined in the bright-field electron transmission microscopy images in Fig. S4(a) contains a domain wall, as detected by means of the stray field modulation visible in the reconstructed phase images. The tilt series acquisition was performed semi-automatically with an in-house developed software to collect two holographic tilt series consisting of object and object-free empty holograms, the second tilt series after the sample was flipped upside-down outside the microscope. For the data shown in Fig. 4 the range of both tilt series (at original and flipped sample) was from −71° to 69° in 2° steps, around the vertical z axis.

To obtain the full phase shift $> 2\pi$, the phase images were unwrapped – automatically by the Flynn algorithm [8], or manually at regions where the phase signal was too noisy or undersampled by using prior knowledge of the phase shift (e.g., from adjacent projections) [9]. Prior to the separation of electric and magnetic phase shifts, the two phase tilt series were processed similarly as reported in Ref. [10].

The resulting tilt series of electrostatic phase images divided by $e/(\hbar v)$ [projected potential V_{proj}, i.e., the first term in Eq. (S9)] was used as input to compute the 3D tomogram of the electrostatic potential by the weighted simultaneous reconstruction technique (WSIRT) algorithm [11]. To reconstruct the internal magnetic state, we removed the stray field outside the nanowire by setting B_z values to zero where the 3D potential V falls below a threshold value of 15 V representing the nanowire surface [Fig. 2(c)].

Following Eq. (S10), the derivatives of the magnetic phase images in the direction *perpendicular* to the tilt axis to obtain the projections of the the axial B_z component times $e/\hbar$. Because the computation of the derivatives enhances noise, the magnetic phase images were smoothed slightly by a low-pass filter (e.g. spatial Gaussian filter). Finally, the WSIRT algorithm was employed to reconstruct the 3D B_z component from the corresponding tilt series.

For visualization and analysis, the tomograms were denoised by means of a non-linear anisotropic diffusion filter within the Avizo software package (ThermoFisher Scientific Company). Fig. S4 depicts the tomographic reconstruction of $B_z(\mathbf{r})$ viewed under two different tilt angles.

Supporting Movies. Visualizing the full 3D reconstructions corresponding to the data shown in Figs. 2 and 4; in all cases the Bloch point position is indicated with a black voxel:

- `3D-pot.mp4`: Volume rendering of the electrostatic potential, corresponding to Fig. 2(d).
- `3D-pot-slicing.mp4` Alternative view on the electrostatic potential of the nanostructure. Left: Showing the surface, corresponding to Fig. 2(c). Right: Cross-sectional slices around the chirality interface, clearly showing the reversal of the helix rotation.
- `3D-Bz.mp4` 3D view of magnetic induction within the nanostructure, corresponding to Fig. 4(c).

Strayfields of Blochpoint and transverse domain walls obtained in phase image tilt series of simulated nanowires. Figure S6 shows the comparison of simulated magnetic phase maps, comparing the observed signatures of Bloch point domain walls and transverse domain walls in magnetic (in our case Co) nanowires. Transverse domain walls are characterised by an asymmetry across the nanowire, which changes with viewing direction. In contrast, Bloch point domain walls exhibit a symmetric magnetic phase contrast that does not depend on the projection direction. This

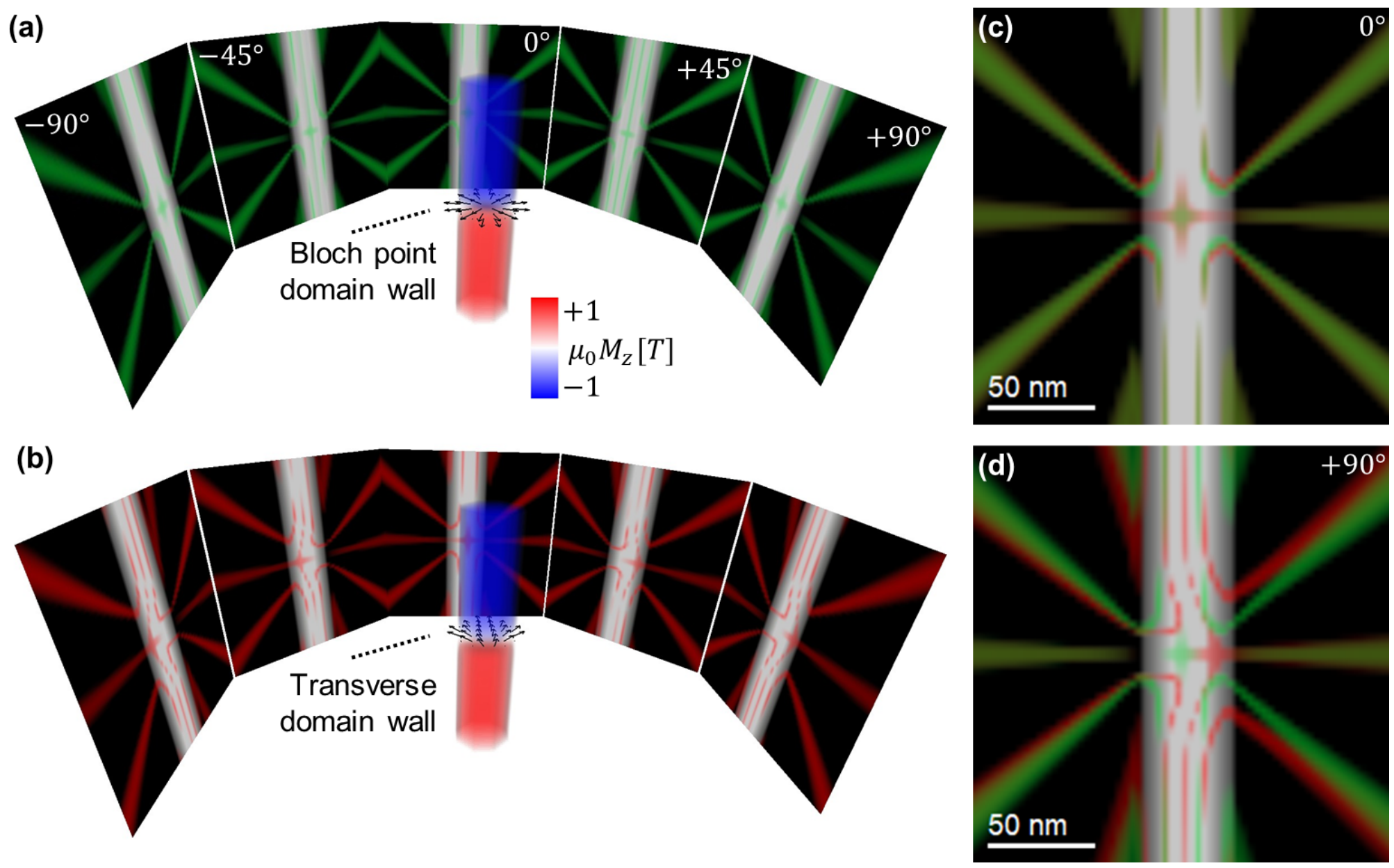

Figure S6: **Stray-fields of Bloch point and transverse domain walls.** (a,b) Simulated phase images of a Co nanowire (42 nm diameter) are shown for various projection angles, depicting a Bloch-point domain wall (a) and a transverse domain wall (b). Electric contributions are rendered in grayscale, while magnetic contributions appear as green iso-lines (as in Fig. 4 of the main text). The stray-field pattern of the Bloch-point wall displays monopole-like symmetry for all viewing directions. In contrast, the transverse wall yields an asymmetric phase image between both sides of the nanowire, with the asymmetry increasing as the angle between the wall's magnetization and the electron beam grows. Phase images extracted from the tilt series at 0° (c) and +90° (d). (c) demonstrates that in both cases Bloch point domain wall (green) and transverse wall (red) the stray field is symmetric, whereas in (d) a pronounced antisymmetry of the stray field for the transverse wall is visible.

supports the main text's claim that, based on the observed symmetry of the stray fields in the phase images, a Bloch point exists (see Fig. 2a,b of the main text).

Triangulation of the Bloch Point position As shown in Fig. S5, the Bloch-point location can be inferred from a specific 2D projection of the magnetic induction. This is accomplished by comparing the projection with magnetic-induction maps calculated from remanent Bloch-point states obtained via micromagnetic simulations. In the experimental data, the Bloch-point appears as features that match the signatures of the projected Bloch point seen in the simulated maps. Combining the determined coordinates from three or more different viewing angles allows triangulation of the Bloch point position in three dimensions.

S5 Alternative Bloch Point Initialization Protocol

Initializing the Bloch point state with the method illustrated in Fig. 1(f,g) requires sizeable fields to be applied; in our case we used at least 0.3 T for the electron holography experiments (Methods Sec. 5.3) and 1.2 T for the x-ray tomography experiments (Methods Sec. 5.2, using lower fields did not lead to a reliable initialization). The magnitude of the initialization fields requires them to be

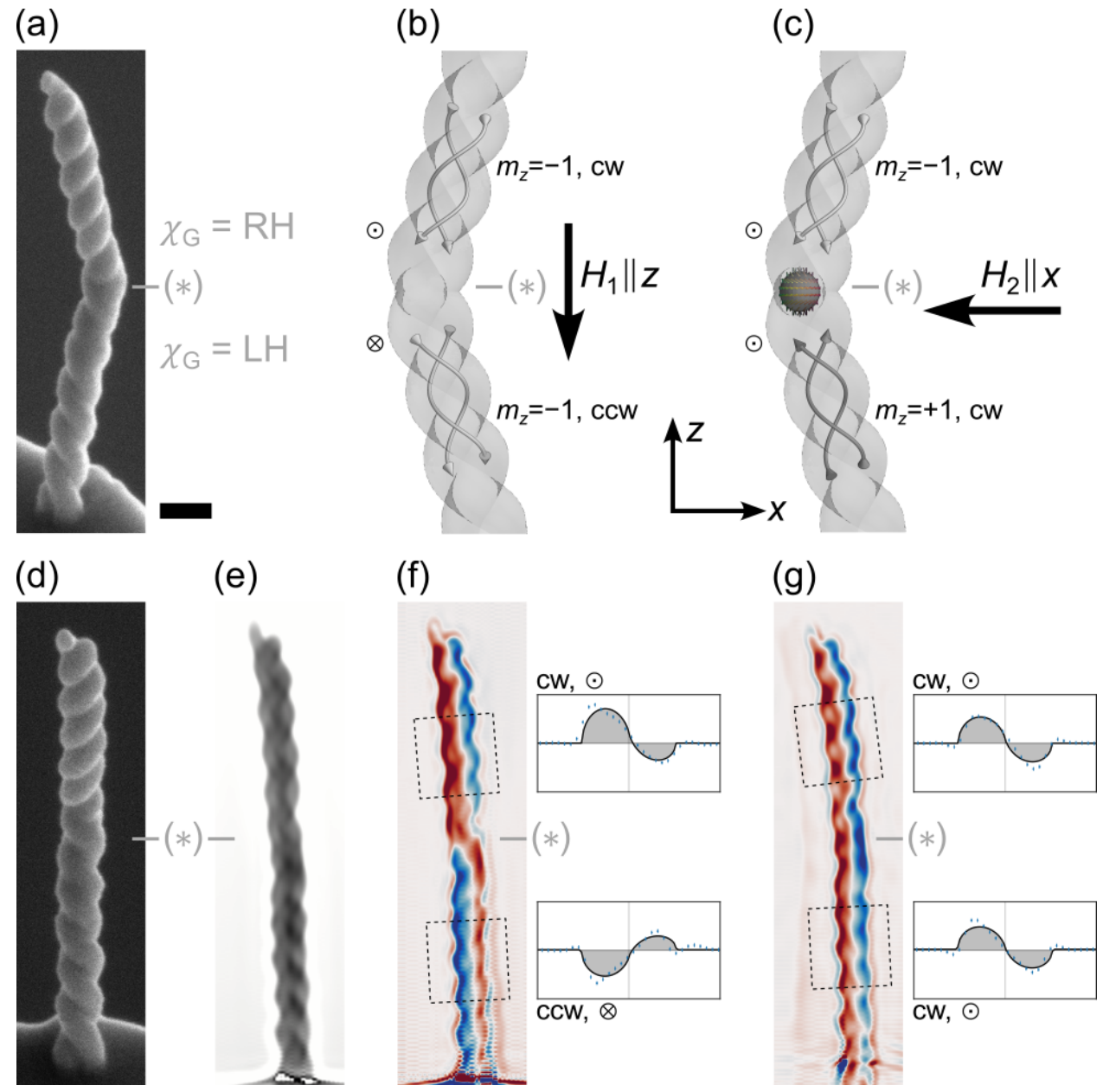

Figure S7: **In-situ two-step initialization of Bloch points.** (a) SEM side view of structure, with the chirality interface marked with (∗). The images were taken under a stage tilt of 52°, and the horizontal scale bar measures 150 nm. (b,c) Schematic illustration of the two-step Bloch point initialization protocol, requiring first to set a well-defined axially-magnetized helical state, followed by simultaneously switching the axial magnetization and circulation in one of the segments by a transverse field. (d,e) Front view of the double-helix structure, measured with (d) SEM and (e) x-ray ptychography. (f) Ptychography XMCD image close to remanence after saturation with $H_1 = 180\,\mathrm{mT} \parallel z$. Boxes indicate regions of interest for which average line profiles (to the right) are extracted. Fits to an analytical model allow to extract the sense of circulation and the axial magnetization into or out of the plane (⊗ and ⊙, respectively). (g) XMCD image at remanence after subsequent application of $H_2 = 180\,\mathrm{mT} \parallel x$, showing a uniform circulation across the entire structure and opposite axial magnetization in each section, indicating the formation of a Bloch point close to the chirality interface.

applied ex-situ, which is undesirable for future experiments, e.g., probing the field- or current-driven motion of Bloch points, where the initial Bloch point state needs to be re-set regularly.

In Fig. S7 we illustrate an alternative two-step protocol to initialize Bloch point domain walls in the helical nanostructure shown in Fig. S7(a). First, after application of an H_z field at remanence an axially magnetized state is set. As shown in Fig. S7(b) the circulation in each segment is opposite, governed by the local condition that $\chi_M = \chi_G$. Then, a transverse field H_x switches the axial magnetization (and subsequently, the circulation) in one of the two legs only, as shown in Fig. S7(c). After relaxation to zero field, the formation of a Bloch-point domain wall close to the chirality interface is expected.

We demonstrate this field protocol using x-ray ptychography and XMCD experiments at the HERMES beamline of the SOLEIL synchrotron. Details of the general experimental approach, image reconstruction and processing can be found elsewhere [5]. Most importantly, a sample environment featuring a tuneable array of permanent magnets [12] allows to apply magnetic fields up to 180 mT along both the beam direction $k \parallel x$, i.e., along the kink of the nanostructure, as well as transverse

to k, i.e., it along the main z nanowire axis.

Figure S7(d,e) shows the front view of the sample both with SEM as well as the structural x-ray contrast of the nanowire, with the chirality interface marked at (∗).

Figure S7(f) shows an XMCD image at $H_z = -10\,\mathrm{mT}$, after application of a field of $H_z = 180\,\mathrm{mT}$, i.e., corresponding to the first step of the field protocol. The transverse red-blue XMCD contrast indicates an opposing helical circulation of magnetization in either half of the structure [5].

We furthermore can fit averaged line cuts of the XMCD contrast (dashed boxes indicate the relevant regions of interest) with an analytical model (black line and shaded area) to extract the sign of the projected axial magnetization m_z as well (see Supporting Information of Ref. [5] for further details). For Fig. S7(f) this projected component points into (⊗) and out of (⊙) the plane on the bottom and top helix, respectively, indicating an overall continuous axial magnetization.

Figure S7(g) shows a subsequent XMCD image at remanence after application of $H_x = 180\,\mathrm{mT}$, i.e., after the second step of the field protocol. Here, a uniform circulation across the entire structure is observed and from the fits we conclude that the axial components of the bottom and top segment both point towards the viewer (⊙). While this observation does not directly probe the local spin structure, it nevertheless indicates the presence of a Bloch-point domain wall, which is expected to form in the vicinity of the chirality interface (∗).

References

(1) Guizar-Sicairos, M.; Thurman, S. T.; Fienup, J. R. Efficient Subpixel Image Registration Algorithms. *Optics Letters* **2008**, *33*, 156–158.

(2) Hierro-Rodriguez, A.; Gürsoy, D.; Phatak, C.; Quirós, C.; Sorrentino, A.; Álvarez-Prado, L. M.; Vélez, M.; Martín, J. I.; Alameda, J. M.; Pereiro, E.; Ferrer, S. 3D Reconstruction of Magnetization from Dichroic Soft X-ray Transmission Tomography. *Journal of Synchrotron Radiation* **2018**, *25*, 1144–1152.

(3) Ruiz-Gómez, S. et al. Tailoring the Energy Landscape of a Bloch Point Domain Wall with Curvature. *Nature Communications* **2025**, *16*, 7422.

(4) Hermosa, J.; Hierro-Rodríguez, A.; Quirós, C.; Martín, J. I.; Sorrentino, A.; Aballe, L.; Pereiro, E.; Vélez, M.; Ferrer, S. Bloch Points and Topological Dipoles Observed by X-ray Vector Magnetic Tomography in a Ferromagnetic Microstructure. *Communications Physics* **2023**, *6*, 49.

(5) Fullerton, J.; Leo, N.; Jurczyk, J.; Donnelly, C.; Sanz-Hernández, D.; Skoric, L.; Mille, N.; Stanescu, S.; MacLaren, D. A.; Belkhou, R.; Hierro-Rodriguez, A.; Fernández-Pacheco, A. Fractional Skyrmion Tubes in Chiral-Interfaced 3D Magnetic Nanowires. *Advanced Functional Materials* **2025**, 2501615.

(6) Hermosa-Muñoz, J.; Hierro-Rodríguez, A.; Sorrentino, A.; Martín, J. I.; Alvarez-Prado, L. M.; Pereiro, E.; Quirós, C.; Vélez, M.; Ferrer, S. Hyperbolic Bloch Points in Ferrimagnetic Exchange Spring. *Results in Physics* **2024**, *61*, 107771.

(7) Tonomura, A.; Matsuda, T.; Endo, J.; Arii, T.; Mihama, K. Holographic Interference Electron Microscopy for Determining Specimen Magnetic Structure and Thickness Distribution. *Physical Review B* **1986**, *34*, 3397–3402.

(8) Ghiglia, D. C. *Two-Dimensional Phase Unwrapping: Theory, Algorithms, and Software*; Wiley: New York, 1998.

(9) Lubk, A.; Wolf, D.; Prete, P.; Lovergine, N.; Niermann, T.; Sturm, S.; Lichte, H. Nanometer-Scale Tomographic Reconstruction of Three-Dimensional Electrostatic Potentials in GaAs/AlGaAs Core-Shell Nanowires. *Physical Review B* **2014**, *90*, 125404.

(10) Wolf, D.; Biziere, N.; Sturm, S.; Reyes, D.; Wade, T.; Niermann, T.; Krehl, J.; Warot-Fonrose, B.; Büchner, B.; Snoeck, E.; Gatel, C.; Lubk, A. Holographic Vector Field Electron Tomography of Three-Dimensional Nanomagnets. *Communications Physics* **2019**, *2*, 87.

(11) Wolf, D.; Lubk, A.; Lichte, H. Weighted Simultaneous Iterative Reconstruction Technique for Single-Axis Tomography. *Ultramicroscopy* **2014**, *136*, 15–25.

(12) Nolle, D.; Weigand, M.; Audehm, P.; Goering, E.; Wiesemann, U.; Wolter, C.; Nolle, E.; Schütz, G. Note: Unique Characterization Possibilities in the Ultra High Vacuum Scanning Transmission x-Ray Microscope (UHV-STXM) "MAXYMUS" Using a Rotatable Permanent Magnetic Field up to 0.22 T. *Review of Scientific Instruments* **2012**, *83*, 046112.
